# Mechanistic Insights Into the Tumor‐Driving and Diagnostic Roles of KCTD Family Genes in Ovarian Cancer: An Integrated In Silico and In Vitro Analysis

**DOI:** 10.1002/cam4.71147

**Published:** 2025-08-20

**Authors:** Ling Zhang, Chong Cheng, Bin Tang

**Affiliations:** ^1^ Centre for Reproductive Medicine, Changde Hospital, Xiangya School of Medicine, Central South University (The First People's Hospital of Changde City) Changde China; ^2^ Department of Nuclear Medicine Changde Hospital, Xiangya School of Medicine, Central South University (The first people’s hospital of Changde city) Changde China

**Keywords:** biomarker, diagnosis, KCTD, miRNA, OC, treatment

## Abstract

**Background:**

Ovarian cancer (OC) remains one of the most lethal gynecological malignancies, characterized by late‐stage diagnosis and high recurrence rates. Despite advances in treatment, the overall survival rate for OC patients remains low due to the lack of reliable biomarkers for early detection and prognosis. Thus, there is an urgent need for novel diagnostic and prognostic biomarkers to improve patient outcomes. In this study, we explored the potential role of the KCTD (Potassium Channel Tetramerization Domain‐containing) family genes in OC.

**Methods:**

This study utilized comprehensive in silico and in vitro experiments.

**Results:**

Firstly, we analyzed the expression patterns of KCTD genes across 12 OC cell lines and 6 normal control cell lines using RT‐qPCR, identifying significant upregulation of KCTD5, KCTD9, KCTD12, and KCTD16, while KCTD2, KCTD10, KCTD15, and KCTD21 were downregulated. ROC analysis revealed high diagnostic accuracy for KCTD2, KCTD5, KCTD9, and KCTD12. Further stage‐specific analysis indicated that KCTD2, KCTD5, KCTD15, and KCTD21 are associated with OC progression. Functional assays in SKOV3 and A2780 cells demonstrated that overexpression of KCTD2 and KCTD10 significantly inhibited cell proliferation, migration, and colony formation, suggesting their tumor‐suppressive roles. Immune and drug sensitivity analyses revealed that KCTD genes may influence immune evasion and chemoresistance in OC. Additionally, miRNA analysis identified potential regulatory mechanisms of KCTD expression.

**Conclusion:**

Collectively, our findings indicate that KCTD family members serve as promising biomarkers, offering new insights into therapeutic strategies for OC management. Further validation in clinical settings is essential to establish their potential as therapeutic targets.

## 1 Introduction

Ovarian cancer (OC) is one of the most lethal gynecological malignancies [1], ranking as the fifth leading cause of cancer‐related death among women [2, 3]. It is often diagnosed at an advanced stage due to the lack of early symptoms and effective screening methods, resulting in a poor prognosis [4, 5, 6]. The majority of OC cases are of the high‐grade serous subtype, which is characterized by rapid progression, frequent recurrence, and resistance to standard therapies [7, 8]. Additionally, genetic alterations such as BRCA1/2 mutations and defects in homologous recombination repair are commonly observed in OC and have both prognostic and therapeutic implications [9]. Despite advances in treatment options such as surgery, chemotherapy, and targeted therapies, the 5‐year survival rate for advanced‐stage OC remains low [10, 11]. This emphasizes the urgent need for reliable diagnostic, prognostic, and therapeutic biomarkers that can aid in early detection, predict patient outcomes, and provide new avenues for treatment. Therefore, studying the molecular mechanisms underlying OC is critical for identifying genes and pathways that drive its progression and for developing novel interventions [12, 13]. Recent integrative studies have highlighted the role of gene expression signatures, DNA methylation patterns, and non‐coding RNAs, such as microRNAs (miRNAs) and long non‐coding RNAs (lncRNAs), in modulating oncogenic pathways in OC [14, 15]. In particular, dysregulated expression of specific miRNAs has emerged as a key factor influencing tumor initiation, metastasis, and chemoresistance, making them attractive candidates for biomarker development and therapeutic targeting [1, 16, 17].

Emerging evidence has highlighted the significance of various gene families in cancer progression [17], including the KCTD family, comprising genes, including KCTD2, KCTD5, KCTD9, KCTD10, KCTD12, KCTD15, KCTD16, and KCTD21 [18, 19, 20]. These genes are involved in essential cellular processes like protein–protein interactions, signal transduction, and apoptosis regulation, all crucial for maintaining cellular homeostasis [21, 22]. Dysregulation of KCTD genes has been observed in various cancer types, indicating their potential role in tumorigenesis. Notably, overexpression of KCTD2 and KCTD3 in breast cancer is associated with poor prognosis and aggressive tumor phenotypes, emphasizing their possible role in promoting tumor growth and metastasis [19, 23]. In colorectal cancer, altered levels of KCTD2 correlate with advanced disease stages and reduced patient survival, suggesting that this gene may drive disease progression and affect patient outcomes [24]. Similarly, in lung cancer, dysregulation of KCTD5 has been linked to increased resistance to chemotherapy, highlighting its role in therapeutic resistance mechanisms and suggesting that targeting KCTD genes may enhance treatment efficacy [20]. Beyond these individual cases, KCTD family genes may act as master regulators of cancer cell behavior by influencing pathways central to cell proliferation, DNA repair, and immune evasion, processes that are often co‐opted by tumors to sustain their growth and evade host defenses [18, 25]. Furthermore, emerging evidence suggests that the KCTD gene family may interact with well‐known oncogenic signaling pathways, such as the PI3K/AKT and MAPK pathways, adding another layer to their potential impact on tumor biology [26, 27]. In the context of diagnostics, the unique expression profiles of KCTD genes across cancer types make them promising biomarkers, potentially aiding in early detection, disease staging, and monitoring of therapeutic response [28, 29].

Despite the growing body of evidence linking KCTD genes to cancer, their roles in OC remain largely unexplored. A few reports have been published so far linking KCTD genes with OC. For example, a study by Engqvist et al. [30] suggested KCTD10 as the histotype‐specific prognostic biomarker of OC. Similarly, another study by Wang et al. [31] discovered KCTD14 as one of the major prognostic biomarkers in OC patients. Given their functional significance in various cancers, it is likely that KCTD genes play a crucial role in the development and progression of OC. However, comprehensive analyses investigating the diagnostic, prognostic, and therapeutic relevance of KCTD family genes in OC are lacking. Therefore, this study aims to fill this knowledge gap by conducting a detailed in silico analysis [32, 33, 34], clinical correlations, and functional roles of these genes; we seek to identify novel biomarkers and potential therapeutic targets that could improve OC management and patient outcomes.

## 2 Methodology

### 2.1 Cell Line Acquisition and Culturing

We obtained a total of 12 commercially available OC cell lines (A2780, CAOV‐3, OVCAR‐3, SKOV‐3, IGROV‐1, HEY, OV‐90, TOV‐21G, ES‐2, COV362, COV318, and PEO1) and 6 normal ovary epithelial cell lines (HOSEpiC, IOSE‐80, T1074, Hs832.Csk, NOEC, and OSE4). These cell lines were selected to represent a broad spectrum of OC subtypes and control samples for comparative analysis. Upon arrival, all cell lines were immediately processed according to ATCC guidelines. The cells were thawed quickly in a 37°C water bath, followed by gentle mixing and subsequent transfer to a sterile 15 mL conical tube containing pre‐warmed complete growth medium. The cells were centrifuged at 200 × g for 5 min; the supernatant was discarded, and the cell pellet was resuspended in fresh growth medium. The OC cell lines were cultured in RPMI‐1640 medium supplemented with 10% fetal bovine serum (FBS), 1% penicillin–streptomycin, and 1% L‐glutamine. The normal ovary cell lines were maintained in DMEM/F‐12 medium supplemented with 10% FBS, 1% penicillin–streptomycin, and 0.5% insulin‐transferrin‐selenium (ITS). All cell lines were grown in a humidified incubator at 37°C with 5% CO_2_. Cells were passaged at 70%–80% confluence using 0.25% trypsin–EDTA for detachment and were seeded at appropriate densities for downstream assays. Cell lines were authenticated using short tandem repeat (STR) profiling to confirm their identity, and mycoplasma testing was performed regularly to ensure cell line integrity and sterility.

### 2.2 RNA Extraction, cDNA Synthesis, and RT‐qPCR Analysis of KCTD Genes

Total RNA was extracted from cell lines using the RNeasy Plus Mini Kit (Qiagen, Hilden, Germany) according to the manufacturer's instructions. Briefly, cells were lysed in the provided lysis buffer, and the lysates were homogenized using QIAshredder columns (Qiagen). RNA was purified, with on‐column DNase treatment to remove genomic DNA, ensuring high‐purity RNA for downstream applications. For cDNA synthesis, 1 μg of total RNA from each sample was reverse transcribed using the High‐Capacity cDNA Reverse Transcription Kit (Applied Biosystems, Foster City, CA, USA) in a 20 μL reaction mixture containing random primers and RNase inhibitor, following the manufacturer's protocol. The reverse transcription reaction was carried out in a thermal cycler at 25°C for 10 min, 37°C for 120 min, followed by 85°C for 5 min to inactivate the reverse transcriptase enzyme.

RT‐qPCR analysis was performed using SYBR Green Master Mix (Thermo Fisher) on a QuantStudio 5 Real‐Time PCR System (Applied Biosystems). Each 10 μL reaction contained 1 μL of cDNA template, 0.5 μL of the primers, 5 μL of Master Mix, and 3.5 μL of nuclease‐free water. Primer efficiency was assessed by generating a standard curve with serially diluted cDNA, and only primers with amplification efficiencies between 90% and 110% and *R* ^2^ values above 0.99 were used. Melt curve analysis was conducted after each reaction to confirm the specificity of the amplified product and rule out non‐specific amplification. The expression levels of each gene were normalized to the endogenous control GAPDH. Relative expression levels were calculated using the 2^−ΔΔCt^ method, and all experiments were conducted in triplicate to ensure reproducibility. Following primers were used to amplify KCTD genes and GAPDH.

GAPDH‐F 5′‐ACCCACTCCTCCACCTTTGAC‐3′

GAPDH‐R 5′‐CTGTTGCTGTAGCCAAATTCG‐3′

KCTD2‐F: 5′‐GCAAACAGCCTGGCTTCATCTG‐3′

KCTD2‐R: 5′‐TTGCTGGCTGAGAGGCATAGTC‐3′

KCTD5‐F: 5′‐TCACGCAGATGGTGTCCACCAT‐3′

KCTD5‐R: 5′‐CAGAGGAACTCGGCTTGGTCTT‐3′

KCTD9‐F: 5′‐GCCGCTGTAATCTTGCACATGC‐3′

KCTD9‐R: 5′‐CAGTTTCAGGGATGCTCCTTCTG‐3′

KCTD10‐F: 5′‐AGACGCTGACCAAGCAGGACAC‐3′

KCTD10‐R: 5′‐AAGTGCTTCCCACAGCGGTCAA‐3′

KCTD12‐F: 5′‐CTTCCGCTACATCCTGGATTACC‐3′

KCTD12‐R: 5′‐AGCTCTGGCAGCTCGAAGTACT‐3′

KCTD15‐F: 5′‐CAGCTCACCAAGTCCAATGCAC‐3′

KCTD15‐R: 5′‐TTCAGTGCCATTGAAGAGGCGG‐3′

KCTD16‐F: 5′‐GCCTGTAACTCATCGGTGACAG‐3′

KCTD16‐R: 5′‐AGCAATCGCAGTGTGAGGGTGA‐3′

KCTD21‐F: 5′‐GCTCAACATCACACTGAACCAGC‐3′

KCTD21‐R: 5′‐GAGGTGCTGAAGATGTTGGCGT‐3′

### 2.3 Validation of KCTD Genes Expression on Extended OC Cohorts

GEPIA2 (http://gepia2.cancer‐pku.cn/) [35] and OncoDB (https://oncodb.org/) [36] are comprehensive online databases for cancer research. GEPIA2 provides gene expression analysis based on RNA sequencing data from thousands of cancer samples, allowing for the exploration of differential expression, survival analysis, and more. OncoDB focuses on cancer‐related gene annotations and provides insights into cancer driver genes and pathways. Both databases were used in this study for the validation of KCTD genes expression using extended cohorts of OC tissue samples from The Cancer Genome Atlas (TCGA).

### 2.4 Mutational, Copy Number Variation (CNV), and Promoter Methylation Analysis of KCTD Genes

cBioPortal (https://www.cbioportal.org/) and GSCA (https://bioinfo.life.hust.edu.cn/GSCA/#/) are powerful cancer genomics databases. cBioPortal provides extensive access to large‐scale cancer datasets, supporting data visualization and clinical analysis, which aids research and personalized medicine [37]. GSCA specializes in gene set enrichment analysis and cancer‐related genomics, offering insights into gene expression, mutation, and pathway alterations across diverse cancers, advancing tumor biology understanding [38]. In this study, the cBioPortal database was utilized for the mutational analysis of KCTD genes, while GSCA was used for the CNV and promoter methylation analysis of KCTD genes in OC.

### 2.5 Prognostic Significance of KCTD Genes

KM Plotter (https://kmplot.com/analysis/) [39, 40] and GENT2 (http://gent2.appex.kr/gent2/) [41] are valuable tools for cancer research. KM Plotter enables survival analysis based on gene expression in multiple cancers, aiding prognosis evaluation. These tools automatically stratify patients into high and low expression groups based on the selected gene's expression level using the median cutoff values. The statistical significance of survival differences between groups is evaluated using the log‐rank test, and results are quantified using hazard ratios (HRs) with 95% confidence intervals (CIs) to estimate the risk associated with gene expression levels. Multivariate analyses, where applicable, can also adjust for confounding clinical variables. Both valuable sources were utilized in this work to explore the prognostic values of KCTD genes in OC.

### 2.6 Correlations of KCTD Genes With Immune Inhibitor and Diverse States of OC

TISIDB (http://cis.hku.hk/TISIDB/) and CancerSEA (http://biocc.hrbmu.edu.cn/CancerSEA/) are specialized cancer immunology databases. TISIDB integrates tumor‐immune interactions, providing resources on immune cells, molecular markers, and immunotherapy targets across cancers [42]. CancerSEA focuses on single‐cell analysis, offering functional states of cancer cells within various tumor types [43]. Together, they facilitate understanding of immune responses and cellular heterogeneity, advancing precision immunotherapy and cancer research. This work utilized both of these databases to explore correlations of KCTD genes with immune inhibitors and diverse states of OC.

### 2.7 miRNA Prediction of KCTD Genes and Their Expression Analysis

TargetScan (https://www.targetscan.org/vert_80/) is a comprehensive database for miRNA target prediction [44]. It identifies miRNA binding sites primarily within the 3′ untranslated regions (3′ UTRs) of target genes and ranks them using a context++ score that considers site accessibility and other contextual features. TargetScan was conducted herein to predict KCTD genes‐related miRNAs. The miRNA prediction analysis identified hsa‐miR‐183‐5p, hsa‐miR‐29c‐3p, hsa‐miR‐218‐5p, hsa‐miR‐9‐5p, hsa‐miR‐4500, hsa‐miR‐199b‐3p, and hsa‐miR‐31‐5p as interacting miRNAs with the KCTD genes. To further investigate the role of these miRNAs, we conducted RT‐qPCR analysis across 12 OC and 6 normal control cell lines.

The RT‐qPCR was performed using pre‐prepared cDNA. Thermo Fisher SYBR Green PCR Master Mix (Catalog No. 4309155) was utilized with specific primers designed for each miRNA: hsa‐miR‐183‐5p (Forward: 5′‐UUUGGCAAUGGUAGAACUCACACU‐3′); hsa‐miR‐29c‐3p (Forward: 5′‐ACACTGCTCCAGGGTAGCACCATTTGAAAT‐3′, Reverse: 5′‐TGGTGTCGTGGAGTCG‐3′); hsa‐miR‐218‐5p (Forward: 5′‐UUGUGCUUGAUCUAACCAUGU‐3′, Reverse: 5′‐AUGGUUAGAUCAAGCACAAUU‐3′); hsa‐miR‐9‐5p (Forward: 5′‐CCGGTCTTTGGTTATCTAGCTG‐3′, Reverse: 5′‐CTCAACTGGTGTCGTGGAGTC‐3′); hsa‐miR‐4500 (Forward: 5′‐UGAGGUAGUAGUUUCUUTAA‐3′, Reverse: 5′‐GCATCAATGGACAACA‐3′); hsa‐miR‐199b‐3p (Forward: 5′‐AACACGCACAGTAGTCTGCA‐3′, Reverse: 5′‐GTCGTATCCAGTGCAGGGT‐3′); and hsa‐miR‐31‐5p (Forward: 5′‐ACACTCCAGCTGGGAGGCAAGATGCTGGC‐3′, Reverse: 5′‐TGGTGTCGTGGAGTCG‐3′). The expression of miRNAs was normalized to U6 snRNA (RNU6B), a commonly used small RNA control for miRNA quantification. The primers for U6 were: Forward: 5′‐CTCGCTTCGGCAGCACA‐3′ and Reverse: 5′‐AACGCTTCACGAATTTGCGT‐3′. The PCR reactions were set up in a 20 μL volume, containing 10 μL SYBR Green PCR Master Mix, 1 μL of forward and reverse primers (10 μM), 2 μL cDNA, and 6 μL nuclease‐free water.

### 2.8 Protein–Protein Interaction (PPI) Network and Gene Enrichment Analysis

STRING (https://string‐db.org/) [45], Genemania (https://genemania.org/) [46], and DAVID (https://david.ncifcrf.gov/) [47] are essential bioinformatics tools for gene and protein interaction analysis. STRING visualizes PPI and functional associations. Genemania predicts gene functions based on interaction networks. DAVID enables functional annotation of genes, supporting insights into pathways and biological processes. Together, these tools enhance understanding of gene relationships and biological functions. We used STRING and Genemania to construct the PPI of KCTD binding partners. Besides this, Venn diagram analysis [48] was conducted to explore common binding partners in PPIs, and DAVID was utilized to conduct gene enrichment analysis.

### 2.9 Inducing Overexpression of KCTD2 and KCTD10 in SKOV‐3 and A2780 Cells

For transfection, KCTD2 and KCTD10 expression plasmids were purchased from VectorBuilder company and transfected using Lipofectamine 3000 reagent (Thermo Fisher, Cat# L3000008) following the manufacturer's protocol. After 48 h, cells were harvested for RNA and protein extraction. RT‐qPCR was performed following the aforementioned protocol. For Western blot, protein lysates were prepared using RIPA buffer (Thermo Fisher, Cat# 89900) with protease inhibitor cocktail (Thermo Fisher, Cat# A32965). Protein concentration was measured with a BCA Protein Assay Kit (Thermo Fisher, Cat# 23225). For Western blot, equal amounts of protein were loaded and separated on an SDS‐PAGE gel, then transferred to PVDF membranes (Millipore, Cat# IPVH00010). Membranes were blocked with 5% BSA in TBST for 1 h, followed by overnight incubation at 4°C with primary antibodies against KCTD2 (Thermo Fisher, Cat# 15553‐1‐AP), KCTD10 (Thermo Fisher, Cat# PA5‐41064), and GAPDH (Thermo Fisher, Cat# PA1‐987). After washing, membranes were incubated with HRP‐conjugated secondary antibodies (Thermo Fisher, Cat# 31460). Bands were visualized using the ECL detection system (Thermo Fisher, Cat# 32106) and analyzed for confirmation of KCTD2 and KCTD10 overexpression.

### 2.10 Cell Proliferation Assay

SKOV‐3 and A2780 ovarian cancer cells were seeded into 96‐well plates at a density of 3000 cells per well in triplicate for each time point. After incubation for 24, 48, and 72 h, cell proliferation was assessed using the Cell Counting Kit‐8 (CCK‐8; Dojindo, Cat# CK04) according to the manufacturer's protocol. Briefly, 10 μL of CCK‐8 reagent was added to each well and incubated for 2 h at 37°C. Absorbance was then measured at 450 nm using a microplate reader to determine cell viability, which reflects the rate of proliferation.

### 2.11 Colony Formation Assay

SKOV‐3 and A2780 ovarian cancer cells were seeded in 6‐well plates at a density of 500 cells per well and cultured under standard conditions (37°C, 5% CO_2_) for 10–14 days, allowing colonies to form. The medium was replaced every 2–3 days to ensure optimal growth conditions. Once visible colonies formed, the wells were gently washed with phosphate‐buffered saline (PBS) and fixed with 4% paraformaldehyde (Thermo Fisher, Cat# 28908) for 20 min at room temperature. After fixation, the cells were washed again with PBS and stained with 0.5% crystal violet solution (Sigma‐Aldrich, Cat# C0775) for 15 min to visualize the colonies. Excess stain was removed by rinsing thoroughly with distilled water, and the plates were air‐dried. Colonies consisting of 30 or more cells were counted manually under a light microscope.

### 2.12 Wound Healing Assay

SKOV‐3 and A2780 cells were seeded in 6‐well plates and grown to near confluence. A scratch was made across the cell monolayer using a sterile 200‐μL pipette tip. Cells were washed with PBS to remove debris and incubated in serum‐free medium. Images of the wound were taken at 0 and 24 h to monitor migration using a phase‐contrast microscope. The percentage of wound closure was calculated to evaluate cell migration.

### 2.13 Statistics

Statistical analyses were performed using GraphPad Prism 9.0 software. To compare differences among more than two experimental groups, a one‐way analysis of variance (ANOVA) was used, which is appropriate for evaluating whether there are statistically significant differences among group means. When ANOVA showed significance, Tukey's post hoc test was applied to account for multiple comparisons and to identify specific group differences. For RT‐qPCR experiments, gene expression levels were quantified using the 2^−ΔΔCt^ method, and comparisons between two independent groups (e.g., treated vs. control) were analyzed using unpaired two‐tailed Student's *t*‐tests, suitable for assessing the difference in means between two normally distributed groups with equal variance. Kaplan–Meier survival analysis was employed to evaluate the prognostic significance of KCTD gene expression in ovarian cancer (OC). Hazard ratios (HRs) and log‐rank *p*‐values were calculated to compare survival distributions between high and low expression groups, which is standard for censored survival data. *p‐value < 0.05, **p‐value < 0.01, and ***p‐value < 0.05 were considered statistically significant.

## 3 Results

### 3.1 Expression Pattern of KCTD Genes in OC

**FIGURE 1:**
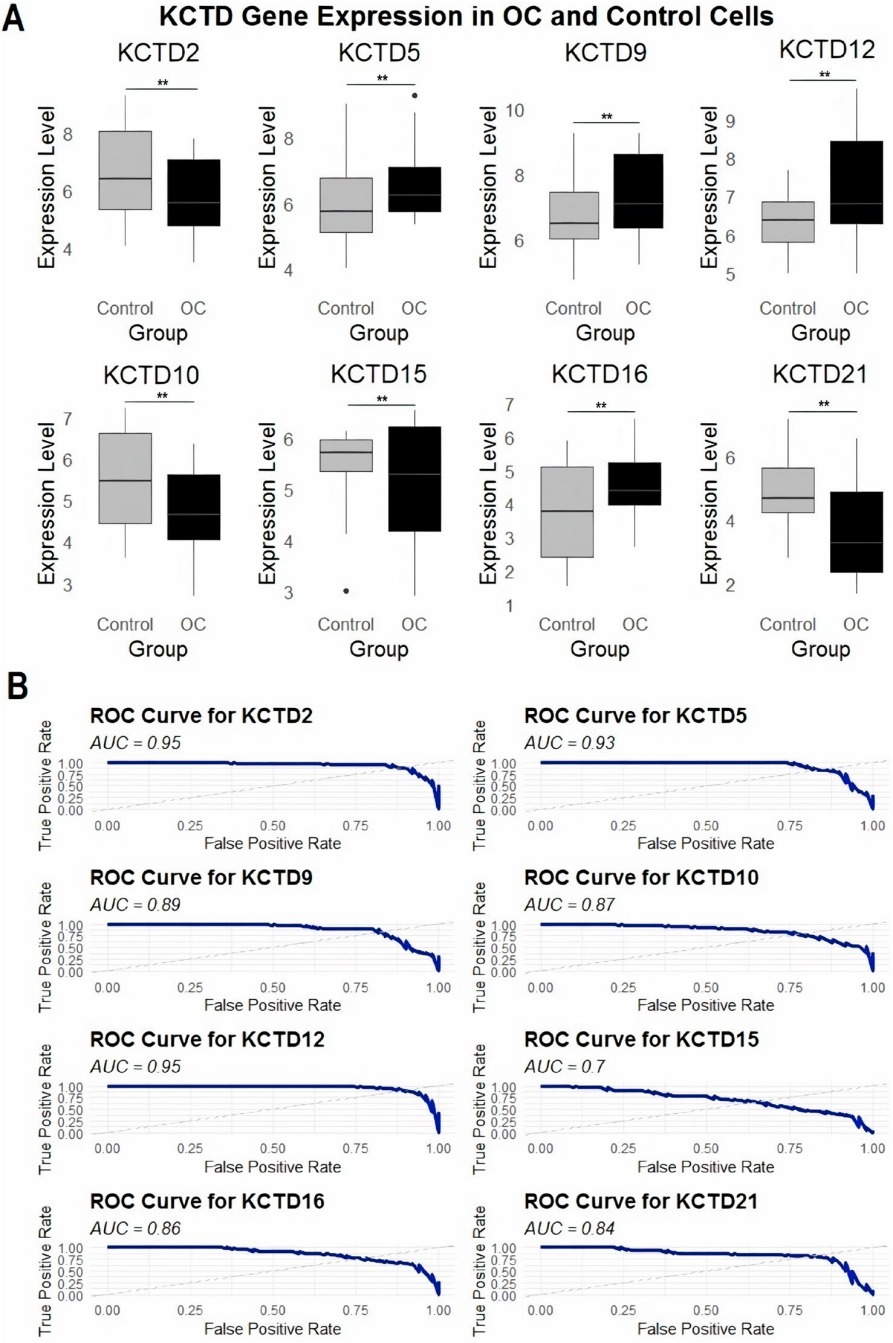
Differential expression and diagnostic performance of KCTD genes in ovarian cancer (OC) cell lines compared to normal ovary epithelial cell lines. (A) RT‐qPCR analysis showing the relative expression levels of selected KCTD genes in 12 ovarian cancer (OC) cell lines and 6 normal control cell lines. KCTD5, KCTD9, KCTD12, and KCTD16 were significantly upregulated in OC cell lines, whereas KCTD2, KCTD10, KCTD15, and KCTD21 were downregulated. (B) Receiver operating characteristic (ROC) curve analysis evaluating the diagnostic efficacy of each KCTD gene in discriminating between OC and normal samples. Notably, KCTD2, KCTD5, KCTD9, and KCTD12 exhibited high diagnostic accuracy with area under the curve (AUC) values ≥ 0.89, suggesting their strong potential as OC biomarkers. In contrast, KCTD10, KCTD15, KCTD16, and KCTD21 demonstrated moderate diagnostic utility with AUCs ranging from 0.70 to 0.87. **p < 0.01.

We analyzed the expression levels of the KCTD genes across 12 OC cell lines compared to 6 normal control cell lines via the RT‐qPCR. Figure 1A presents the expression levels of KCTD genes, where KCTD5, KCTD9, KCTD12, and KCTD16 were significantly upregulated in OC cell lines compared to controls, while KCTD2, KCTD10, KCTD15, and KCTD21 showed decreased expression in OC cell lines. Furthermore, Figure 1B illustrates the ROC curves for each KCTD gene to evaluate their diagnostic efficacy. Area under the curve (AUC) values for KCTD2 (0.95), KCTD5 (0.93), KCTD9 (0.89), and KCTD12 (0.95) indicate a high diagnostic accuracy, highlighting these genes as promising markers for OC (Figure 1B). In contrast, KCTD10, KCTD15, KCTD16, and KCTD21 have lower AUC values, ranging from 0.7 to 0.87, suggesting relatively moderate diagnostic potential (Figure 1B).

### 3.2 Validation of KCTD Genes Expression in Using Extended Cohorts of OC Tissue Samples From TCGA

**FIGURE 2:**
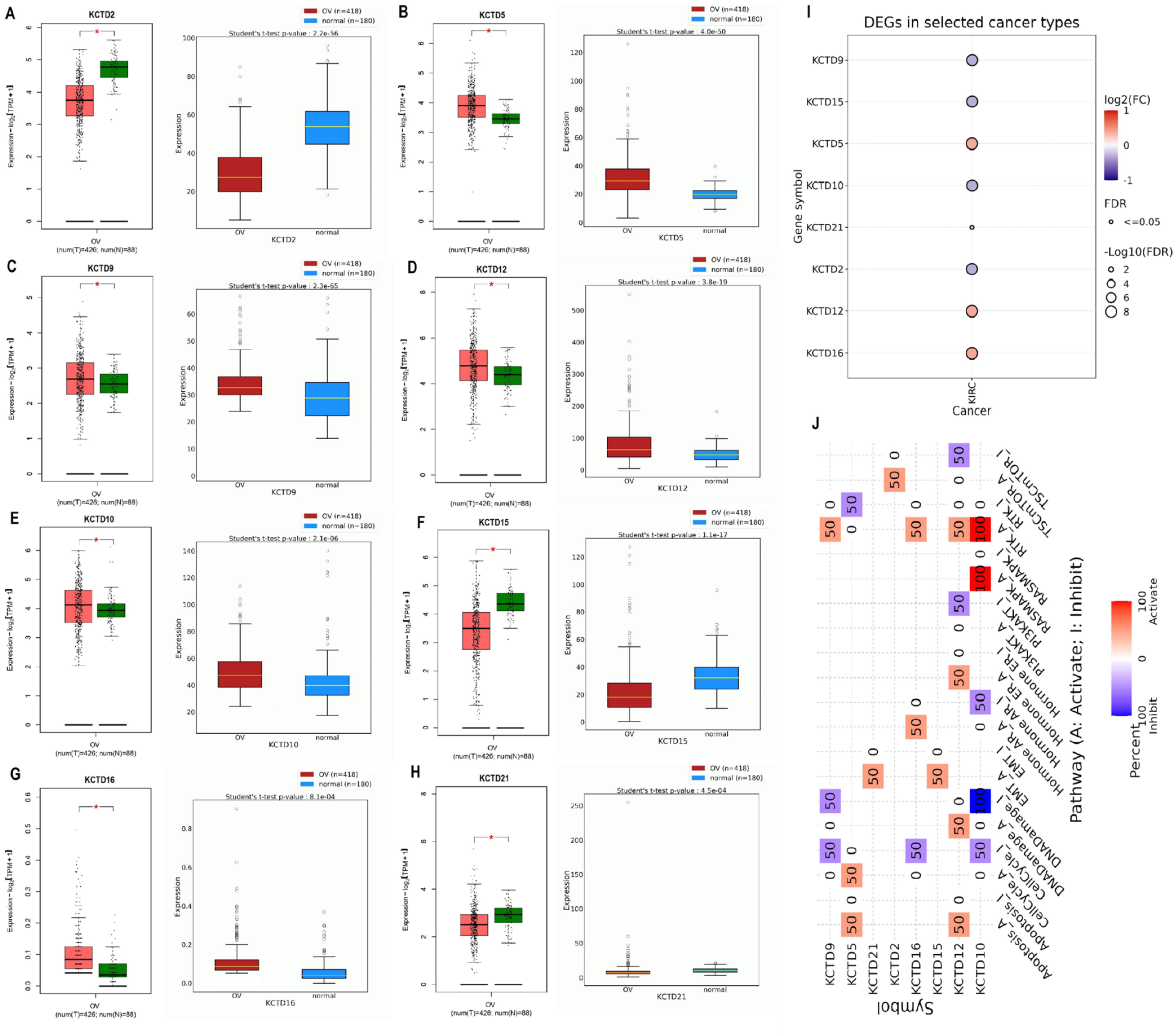
Validation of KCTD gene expression and analysis of cancer‐related cellular processes in ovarian cancer (OC) tissues samples. (A–I) Differential expression analysis of KCTD2, KCTD5, KCTD9, KCTD10, KCTD12, KCTD15, KCTD16, and KCTD21 genes between ovarian cancer (OC) tissues and normal ovarian tissues using multiple large‐scale datasets: GEPIA2 (88 normal vs. 426 OC samples), OncoDB (180 normal vs. 418 OC samples), and GSCA databases. Consistent with RT‐qPCR results in cell lines, KCTD5, KCTD12, and KCTD16 were significantly upregulated, while KCTD2, KCTD10, KCTD15, and KCTD21 were downregulated in OC tissues. A notable exception was KCTD9, which showed high expression in OC cell lines but was significantly reduced in tissue samples, indicating potential tissue‐specific regulation or context‐dependent expression. (J) Functional enrichment analysis from the GSCA database illustrating the association of dysregulated KCTD genes with cancer‐related cellular processes. The heatmap highlights their potential roles in regulating pathways such as cell proliferation, apoptosis, DNA repair, and immune responses, suggesting their contribution to OC tumorigenesis and progression. *p < 0.01.

Next, we validated the expression of KCTD family genes in OC tissues and investigated their potential roles in cancer‐related cellular processes. Figure 2A–I showed the differential expression of KCTD2, KCTD5, KCTD9, KCTD10, KCTD12, KCTD15, KCTD16, and KCTD21 between OC tissues and normal tissues, based on data from an extended cohort of OC samples in TCGA via the GEPIA2 (Containing 88 normal samples and 426 OC samples), OncoDB (Containing 180 normal samples and 418 OC samples), and GSCA databases. Results revealed that KCTD5, KCTD12, and KCTD16 were significantly upregulated in OC tissues, while KCTD2, KCTD9, KCTD10, KCTD15, KCTD16, and KCTD21 were significantly downregulated in OC cell lines (Figure 2A–I). Validation analysis confirmed the results obtained from OC cell lines for all genes, except for KCTD9, which was overexpressed in OC cell lines but significantly downregulated in OC tissue samples. Finally, Figure 2J, obtained from the GSCA database, presents the impact of these dysregulated KCTD genes on various cellular processes in OC patients, indicating that these genes play roles in both activating and inhibiting pathways critical to cancer progression, such as cell proliferation, apoptosis, and DNA repair. Overall, these findings strengthen the evidence that specific KCTD family members are significantly altered in OC and may contribute to tumor development and progression by modulating key cellular mechanisms.

### 3.3 Expression of KCTD Genes Across Different Stages of OC and Performing Mutational Analysis

**FIGURE 3:**
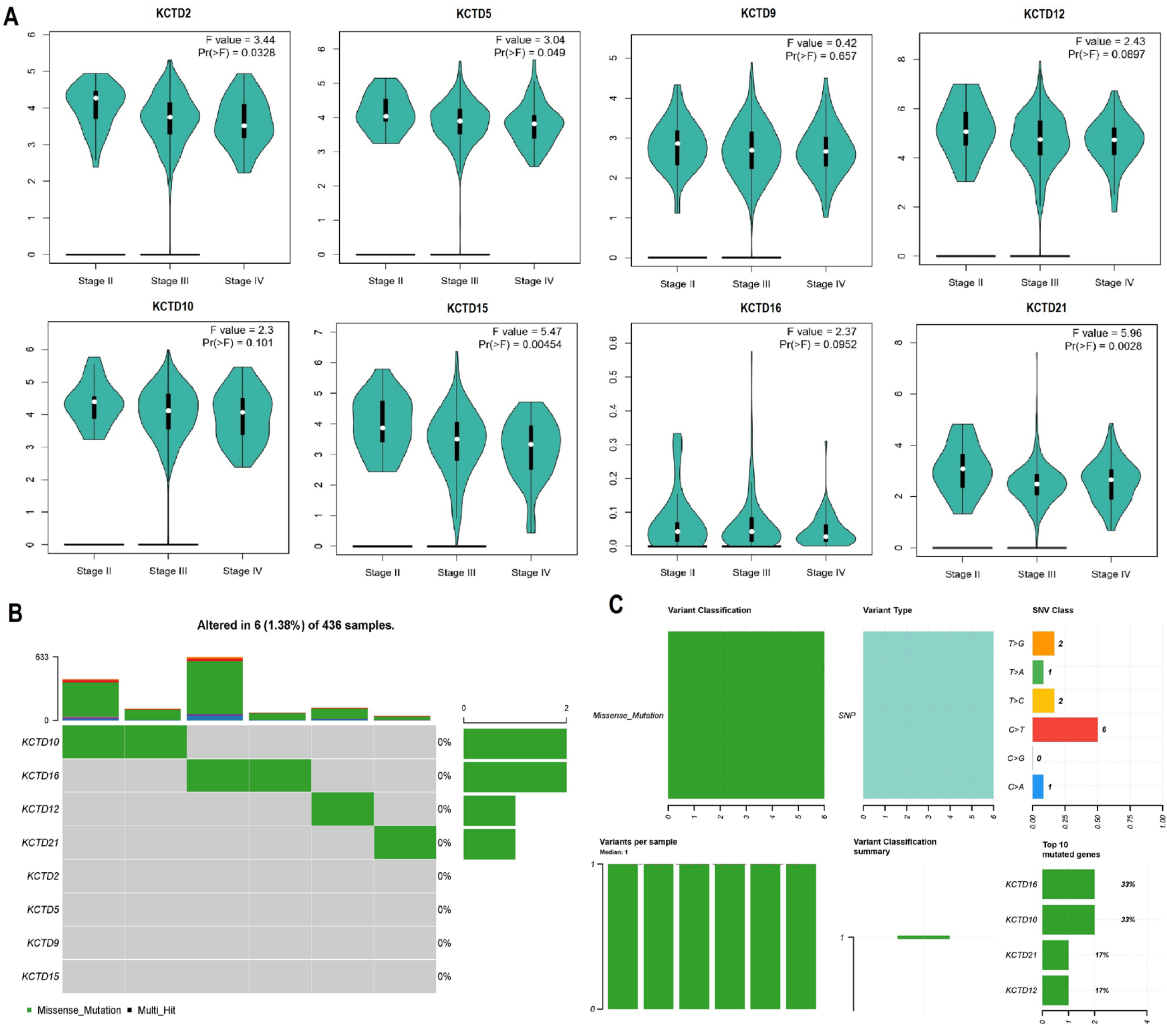
Expression profiles and mutational analysis of KCTD family genes in ovarian cancer (OC) stages. (A) Expression patterns of KCTD2, KCTD5, KCTD9, KCTD10, KCTD12, KCTD15, KCTD16, and KCTD21 across OC stages II, III, and IV based on GEPIA2 database analysis. (B) Mutational analysis of KCTD family genes in OC from the cBioPortal database. (C) Variant type distribution of KCTD mutations, indicating that missense mutations are the most common, with single nucleotide polymorphisms (SNPs) predominantly present. *p* < 0.01.

This section of our study evaluated the expression and mutational profiles of the KCTD family genes across different OC stages to understand their potential roles in OC progression and mutation patterns. Figure 3A shows the expression patterns of KCTD2, KCTD5, KCTD9, KCTD10, KCTD12, KCTD15, KCTD16, and KCTD21 across OC stages II, III, and IV, based on data from GEPIA2. Notably, KCTD2 and KCTD5 exhibited significant differential expression with *F*‐values of 3.44 (*p* = 0.0328) and 3.04 (*p* = 0.049), respectively, suggesting a potential association with OC progression (Figure 3A). KCTD15 and KCTD21 also displayed significant expression differences, with KCTD15 showing an *F*‐value of 5.47 (*p* = 0.00454) and KCTD21 an *F*‐value of 5.96 (*p* = 0.0028) (Figure 3A). However, KCTD9, KCTD10, KCTD12, and KCTD16 did not exhibit statistically significant changes across stages, indicating that their expression might not be stage‐dependent (Figure 3A). Furthermore, Figure 3B shows the mutational analysis results of the KCTD family genes in OC, using data from the cBioPortal database. Among 436 samples, mutations were identified in a small subset (6 samples or 1.38%) (Figure 3B), with KCTD10, KCTD16, KCTD12, KCTD21, KCTD5, KCTD9, and KCTD15 showing missense mutations or multi‐hit events, though at low frequencies. Figure 3C provides further details on the types of variants observed. Most mutations were missense mutations, with single nucleotide polymorphisms (SNPs) as the predominant variant type. The SNV classification plot shows that C>T and C>A transitions are the most frequent changes among the variants (Figure 3C).

### 3.4 Promoter Methylation and CNV Analysis of KCTD Genes in OC

**FIGURE 4:**
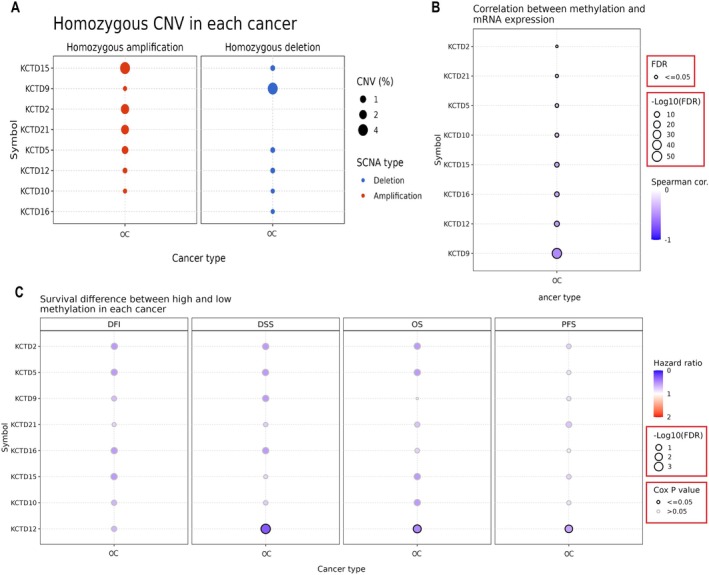
Analysis of copy number variation (CNV), promoter methylation, and survival impact of KCTD family genes in ovarian cancer (OC). (A) CNV analysis of KCTD genes in OC, showing levels of homozygous amplification and deletion based on GSCA database data. (B) Correlation between promoter methylation and mRNA expression levels for KCTD genes in OC via GSCA database, (C) Impact of promoter methylation on survival outcomes of OC patients. *p* < 0.05 and FDR < 0.05.

Next, we conducted analyses of CNV, promoter methylation, and the impact of methylation on survival outcomes for KCTD family genes in OC using the GSCA database. Figure 4A displays the CNV analysis of KCTD genes, showing both homozygous amplifications and deletions in OC. Notably, KCTD15, KCTD2, KCTD21, and KCTD5 exhibit higher levels of homozygous amplification, while KCTD9, KCTD12, KCTD10, and KCTD16 show homozygous deletions (Figure 4A). Moreover, Figure 4B illustrates the correlation between promoter methylation and mRNA expression levels for KCTD genes in OC. Significant (FDR < 0.05) negative correlations were observed between the expression and promoter methylation levels of KCTD2, KCTD5, KCTD9, KCTD10, KCTD12, KCTD15, KCTD16, and KCTD21 in OC (Figure 4B). Additionally, Figure 4C illustrates the impact of methylation levels on various survival outcomes in ovarian cancer (OC) patients, including disease‐free interval (DFI), disease‐specific survival (DSS), overall survival (OS), and progression‐free survival (PFS). Notably, among the KCTD genes, only KCTD12 shows a statistically significant association (*p*‐value < 0.05) with DSS, OS, and PFS, where lower methylation levels are linked to poorer survival outcomes. In contrast, the other KCTD genes display no significant correlations. To ensure the reliability of these findings, corrections for multiple comparisons were applied.

### 3.5 Prognostic Significance of KCTD Genes in OC

**FIGURE 5:**
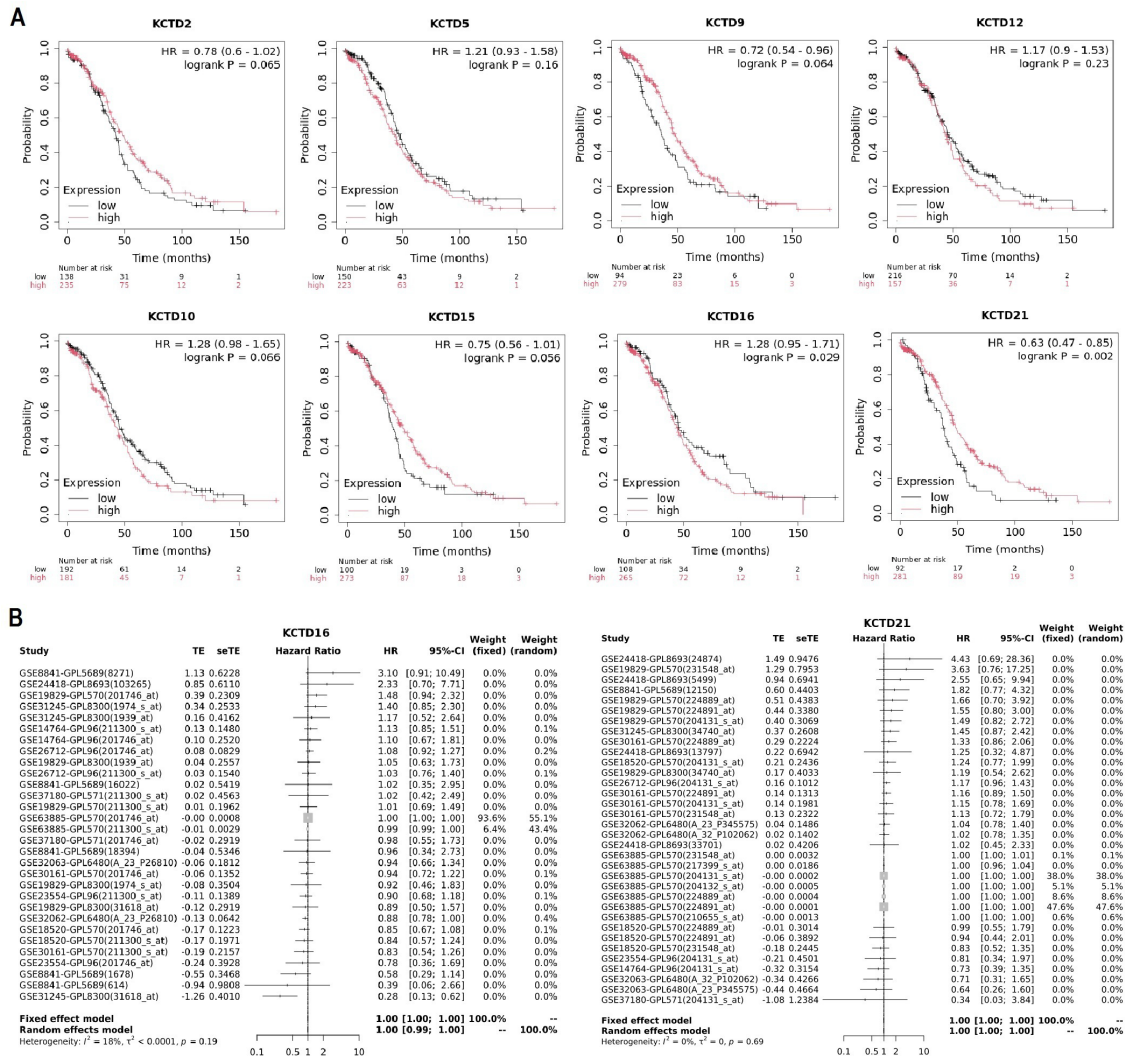
Prognostic significance of KCTD family gene expression in ovarian cancer (OC) patients. (A) Kaplan–Meier survival curves showing the association between expression levels of selected KCTD family genes (KCTD2, KCTD5, KCTD9, KCTD10, KCTD12, KCTD15, KCTD16, and KCTD21) and overall survival (OS) in OC patients, analyzed using the Kaplan–Meier Plotter database. High expression of KCTD16 (HR = 1.28, *p* = 0.029) and KCTD21 (HR = 0.63, *p* = 0.002) was significantly associated with worse OS. (B) Forest plot analysis from the GENT2 database summarizing hazard ratios (HRs) and 95% confidence intervals (CIs) for KCTD16 and KCTD21 across multiple studies. Pooled results from fixed and random effects models indicate no significant association between gene expression and OS (HR = 1.00, 95% CI: 0.99–1.01). *p* < 0.01.

We evaluated the prognostic significance of KCTD family genes in OC by analyzing their impact on patients' overall survival through Kaplan–Meier plotter and GENT2 datasets. Figure 5A presents survival curves for KCTD2, KCTD5, KCTD9, KCTD10, KCTD12, KCTD15, KCTD16, and KCTD21, generated using KM plotter. The results indicate that high expression levels of KCTD16 (HR = 1.28, *p* = 0.029) and KCTD21 (HR = 0.63, *p* = 0.002) were associated with worst overall survival in OC patients (Figure 5A). However, for other KCTD family members, the differences in survival between high and low expression groups were not statistically significant, implying a less clear role in OC prognosis. Furthermore, Figure 5B provides a forest plot from the GENT2 database for KCTD16 and KCTD21, summarizing hazard ratios (HRs) and confidence intervals (CIs) across multiple studies. The fixed and random effects models show a pooled HR of 1.00 (95% CI: 0.99–1.01) for both KCTD16 and KCTD21, indicating no significant association between their expression and overall survival across the included studies (Figure 5B). Overall, these analyses suggest that KCTD9 and KCTD21 may have unfavorable prognostic implications in OC, while the results for KCTD16 remain inconclusive. Further validation in larger, independent cohorts is necessary to clarify the role of KCTD family members as prognostic biomarkers in OC.

### 3.6 Correlations of KCTD Genes With Immune Inhibitor and Diverse States of OC

**FIGURE 6:**
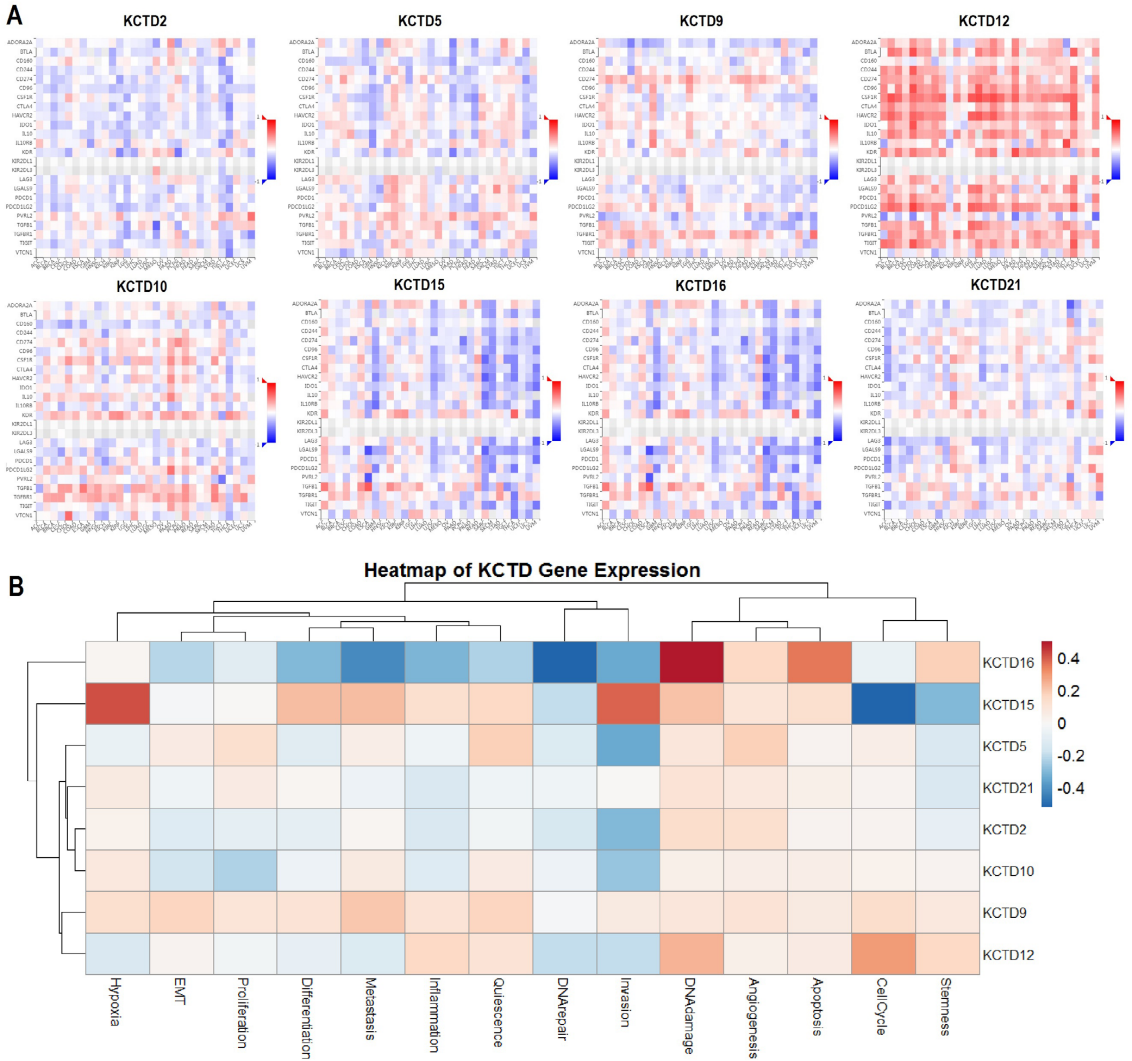
Correlation of KCTD family genes with immune inhibitors and oncogenic states in ovarian cancer (OC). (A) Correlation of KCTD family genes with immune inhibitors in OC, analyzed using the TISIDB database. (B) Correlation of KCTD family genes with oncogenic states in OC, analyzed using the CancerSEA database. *p* < 0.01.

In this section, we analyzed the correlation of specific KCTD genes with immune inhibitors in OC using the TISIDB database, and with various oncogenic states using the CancerSEA database. In Figure 6A, KCTD genes were found correlating with important immune inhibitors. For example, KCTD12 shows a strong positive correlation with several immune inhibitors, including CD244, CSF1R, CTLA‐4, IL10, and TGFB1 (Figure 6A). This suggests that KCTD genes may contribute to immune suppression in OC, potentially aiding in tumor immune evasion. Moreover, Figure 6B illustrates the correlation of KCTD genes with diverse oncogenic states in OC. Results showed that KCTD16 was moderately positively correlated with hypoxia, proliferation, DNA damage, and stemness, suggesting its involvement in promoting these tumorigenic processes (Figure 6B). KCTD15 showed strong positive correlations with hypoxia and invasion, highlighting its potential role in enhancing these cancer‐related functions (Figure 6B). Similarly, KCTD12 was also positively correlated with DNA damage and cell cycle (Figure 6B).

### 3.7 miRNA Prediction of KCTD Genes and Their Expression Analysis

**FIGURE 7:**
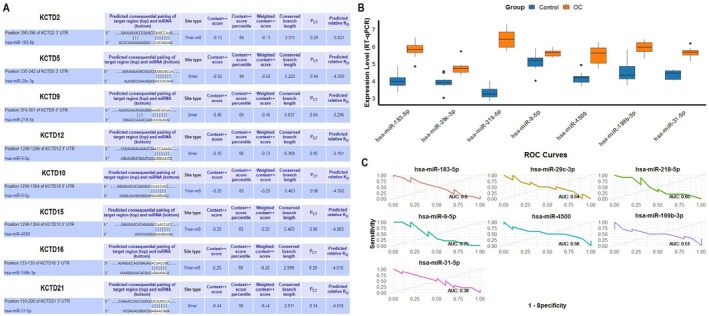
Predicted regulatory miRNAs of KCTD genes and their diagnostic potential in ovarian cancer (OC). (A) Predicted miRNAs regulating KCTD genes, identified using the TargetScan database. (B) Expression levels of the predicted miRNAs in OC and control cell lines, analyzed via RT‐qPCR. (C) Diagnostic potential of the predicted miRNAs, evaluated through ROC curve analysis. *p* < 0.01.

Next, we analyzed the predicted regulatory miRNAs of KCTD genes using the TargetScan database, their expression levels in OC and control cell lines, and their diagnostic potential using ROC curves. Figure 7A shows the predicted miRNAs that regulate KCTD gene expression. Specific miRNAs, including hsa‐miR‐183‐5p (for KCTD2), hsa‐miR‐29c‐3p (for KCTD5), hsa‐miR‐218‐5p (for KCTD9), hsa‐miR‐9‐5p (for KCTD12 and KCTD12), hsa‐miR‐4500 (for KCTD15), hsa‐miR‐199b‐3p (for KCTD16), and hsa‐miR‐31‐5p (for KCTD21), were identified as potential regulators based on target pairing within the 3′ UTR of each KCTD gene using the TargetScan database. Expression analysis of the predicted miRNAs was carried out across 12 OC and 6 normal control cell lines using RT‐qPCR. Results of the analysis showed that the hsa‐miR‐183‐5p, hsa‐miR‐29c‐3p, hsa‐miR‐218‐5p, hsa‐miR‐9‐5p, hsa‐miR‐4500, hsa‐miR‐199b‐3p, and hsa‐miR‐31‐5p miRNAs were significantly (*p*‐value < 0.05) upregulated in the OC cell line group compared to the control cell lines (Figure 7B). This increased expression suggests these miRNAs may play a role in OC by potentially regulating their respective KCTD gene targets. Figure 7C further presents the ROC curves to evaluate the diagnostic potential of these miRNAs in distinguishing OC from control cell lines. The ROC analysis reveals that hsa‐miR‐183‐5p (AUC: 0.8), hsa‐miR‐9‐5p (AUC: 0.75), and hsa‐miR‐218‐5p (AUC: 0.65) show relatively higher AUC values, suggesting better diagnostic potential for OC. In contrast, hsa‐miR‐29c‐3p (AUC: 0.54), hsa‐miR‐4500 (AUC: 0.56), and hsa‐miR‐199b‐3p (AUC: 0.51) have moderate diagnostic value, while hsa‐miR‐31‐5p (AUC: 0.38) exhibits low diagnostic potential (Figure 7C).

### 3.8 PPI Network Construction and Gene Enrichment Analyses

**FIGURE 8:**
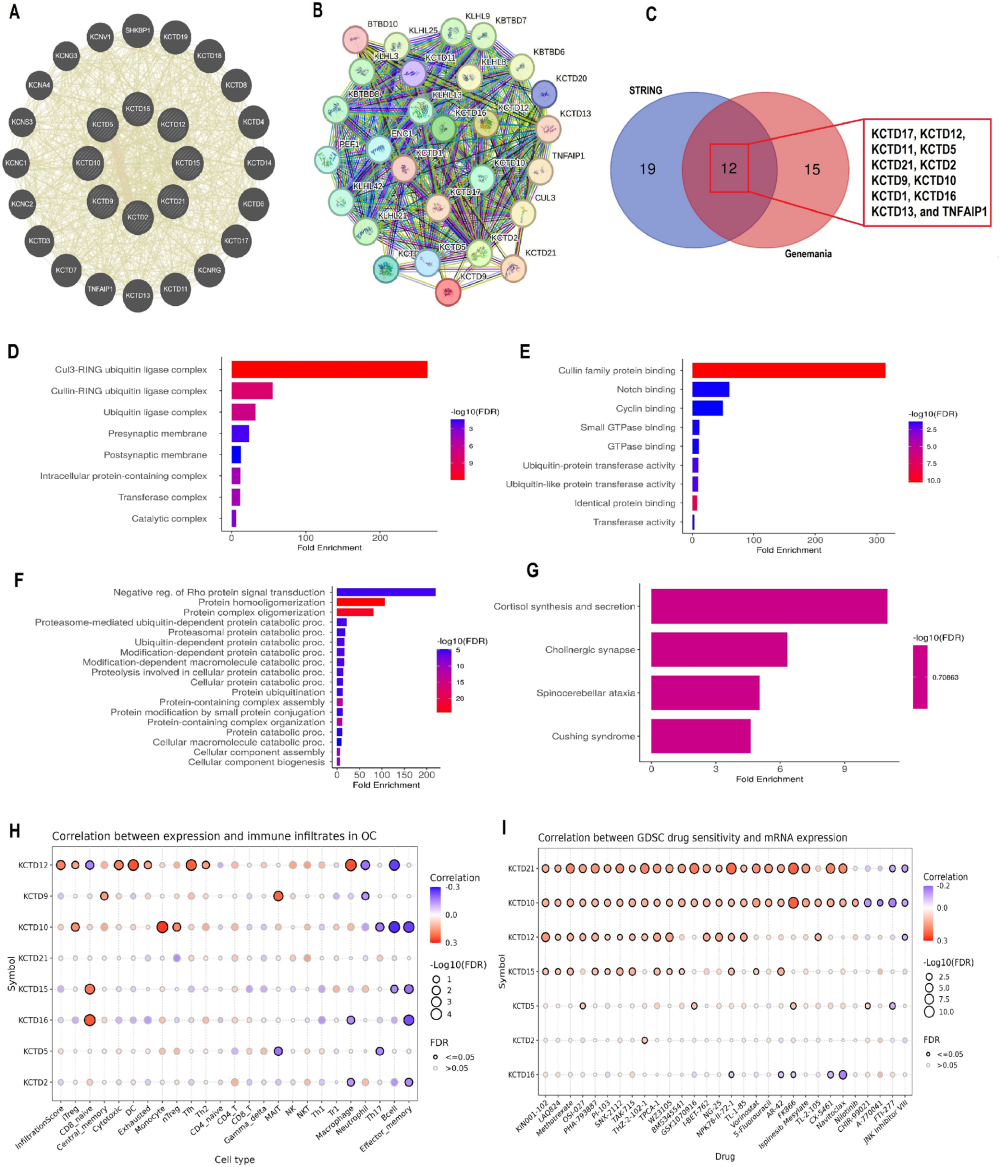
PPI network, functional enrichment, immune infiltration, and drug sensitivity analysis of KCTD family genes in ovarian cancer. (A) PPI network of KCTD family genes generated using Genemania. (B) PPI network of KCTD proteins created using the STRING database. (C) Venn diagram depicting common binding partners identified in both Genemania and STRING PPI networks. (D) Cellular components analysis. (E) Molecular function analysis. (F) Biological process analysis. (G) Pathway analysis. (H) Immune infiltration analysis showing the correlation between KCTD gene expression and various immune cell types in the OC tumor microenvironment, based on data from the GSCA database. (I) Drug sensitivity analysis of KCTD genes using the GDSC database. *p* < 0.01.

In this section of our study, we performed a comprehensive PPI network, functional enrichment analysis, immune infiltration analysis, and drug sensitivity analysis to explore the interacting partners, biological pathways, and potential clinical relevance of KCTD family genes in OC. Figure 8A displays the PPI network of KCTD family genes generated using Genemania, which illustrates interactions between various KCTD family members. Figure 8B shows a similar PPI network created using the STRING database, presenting a broader view of interactions among KCTD proteins and other associated proteins. Furthermore, Venn diagram analysis was conducted in Figure 8C to explore common binding partners between both PPIs created via Genemania and STRING database. Results revealed 12 common binding partners across both networks: KCTD17, KCTD12, KCTD11, KCTD5, KCTD21, KCTD2, KCTD9, KCTD10, KCTD1, KCTD16, KCTD13, and TNFAIP1. These 12 binding partners were further subjected to gene enrichment analysis using DAVID. Figure 8D shows their involvement in cellular components such as the “Cul3‐RING ubiquitin ligase complex,” suggesting a role in protein degradation. Figure 8E revealed molecular functions related to “Cullin family protein binding,” “Notch binding,” and “Cyclin binding.” Further biological process analysis results in Figure 8F identify processes like “protein homooligomerization and protein complex homooligomerization. Figure 8G suggests potential associations of these genes with pathways in “cortisol synthesis and secretion,” “cholinergic synapse,” and “Cushing syndrome.”

Immune infiltration analysis results in Figure 8H illustrated the correlation between KCTD gene expression and various immune cells in OC using GSCA database. Results showed specific correlations with different immune cell types, suggesting that certain KCTD genes might influence the immune environment within the tumor microenvironment (Figure 8H). For example, KCTD12 was positively correlated with the infiltration of Th2 cells and DC cells, suggesting a potential role in modulating immune responses within the tumor microenvironment (Figure 8H). Similarly, KCTD5 shows a positive correlation with monocyte, nTreg, monocytes, and iTreg cells, hinting at an involvement in innate immune activity (Figure 8H). Furthermore, Figure 8I present the correlations between the expression of KCTD genes and drug sensitivity in the Genomics of Drug Sensitivity in Cancer (GDSC) database. This analysis identified several significant relationships. For instance, KCTD12 expression was positively correlated with to LAQ824, suggesting that higher KCTD12 levels may enhance resistance to this drug (Figure 8I).

### 3.9 Inducing Overexpression of KCTD2 and KCTD10 in OC Cells and Performing Functional Analysis

**FIGURE 9:**
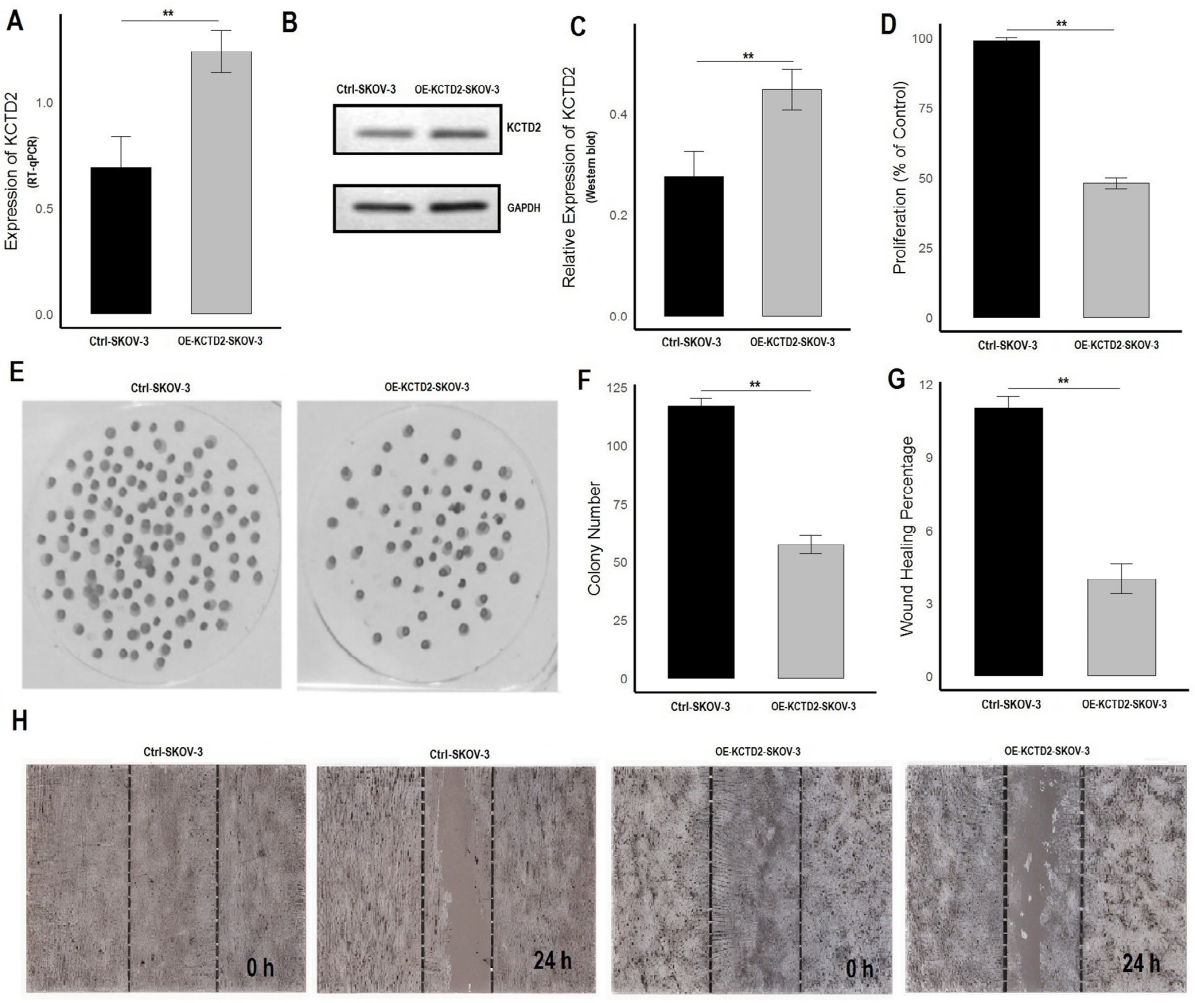
Functional analysis of KCTD2 overexpression in SKOV3 cells. (A) RT‐qPCR analysis showing significant upregulation of KCTD2 mRNA expression in OE‐KCTD2‐SKOV3 cells compared to control SKOV3 cells (Ctrl‐SKOV3). (B) Western blot analysis confirming the overexpression of KCTD2 protein in OE‐KCTD2‐SKOV3 cells. (C) Relative expression of KCTD2 protein as measured by Western blot. (D) CCK‐8 assay results demonstrating a significant reduction in cell proliferation in OE‐KCTD2‐SKOV3 cells compared to Ctrl‐SKOV3 cells. (E, F) Colony formation assay showing a decrease in colony number and size in OE‐KCTD2‐SKOV3 cells, indicating reduced clonogenic potential compared to Ctrl‐SKOV3 cells. (G–H) Wound healing assay illustrating impaired migratory ability in OE‐KCTD2‐SKOV3 cells, with a significantly reduced wound closure compared to Ctrl‐SKOV3 cells, suggesting a role of KCTD2 in inhibiting cell migration. **p < 0.01.

**FIGURE 10:**
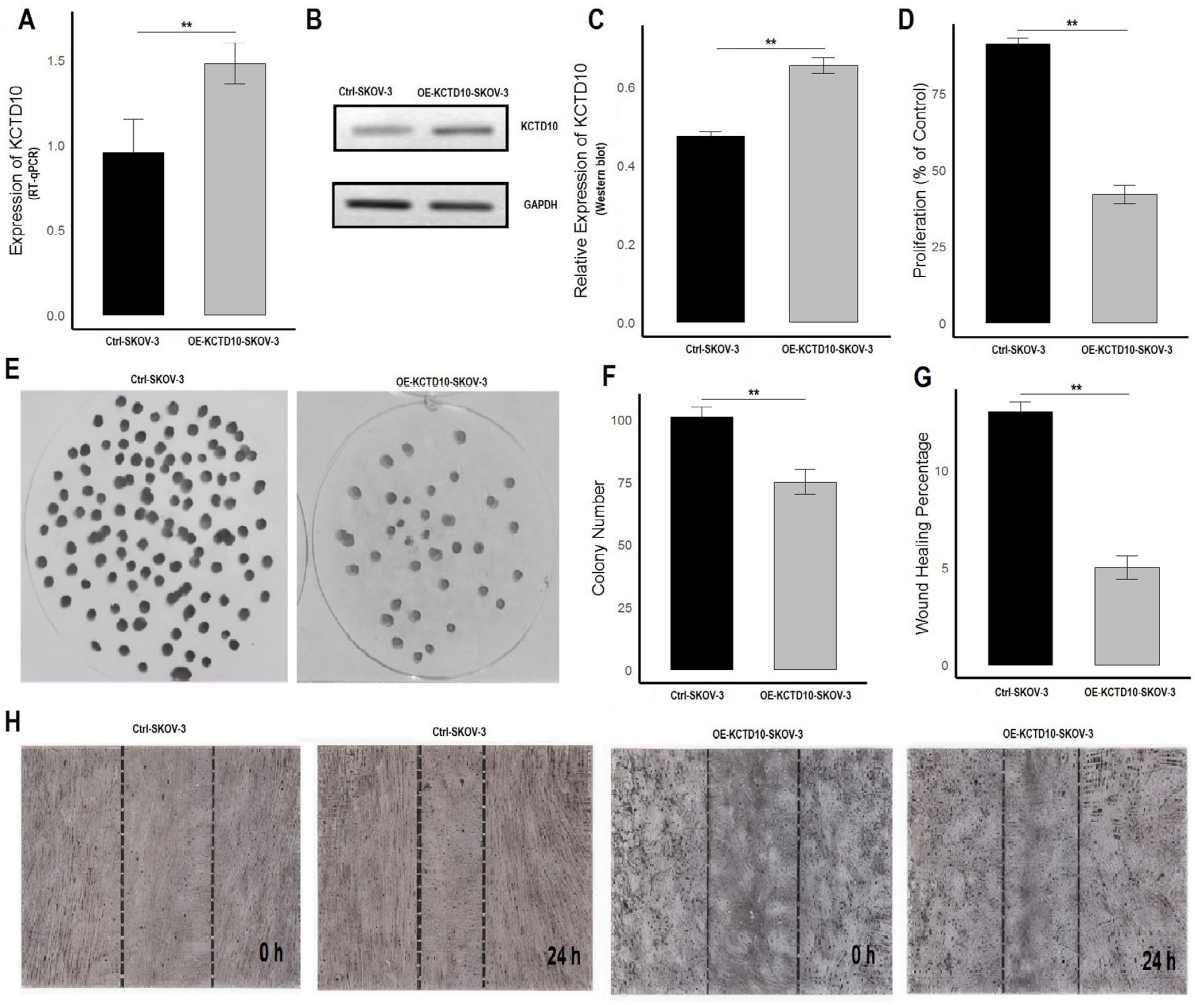
Functional analysis of KCTD10 overexpression in SKOV3 cells. (A) RT‐qPCR analysis revealing a significant increase in KCTD10 mRNA expression in OE‐KCTD10‐SKOV3 cells relative to control SKOV3 cells (Ctrl‐SKOV3). (B) Western blot analysis confirming the elevated expression of KCTD10 protein in OE‐KCTD10‐SKOV3 cells. (C) Relative expression of KCTD10 protein as measured by Western blot. (D) CCK‐8 assay results showing a marked decrease in cell proliferation in OE‐KCTD10‐SKOV3 cells compared to Ctrl‐SKOV3 cells. (E, F) Colony formation assay demonstrating a reduction in both the number and size of colonies in OE‐KCTD10‐SKOV3 cells, indicating diminished clonogenic capacity compared to Ctrl‐SKOV3 cells. (G–H) Wound healing assay displaying a significant impairment in migration in OE‐KCTD10‐SKOV3 cells, with slower wound closure compared to Ctrl‐SKOV3 cells, suggesting that KCTD10 inhibits cell migration. **p < 0.01.

In the final part of our study, we elucidated the functional roles of KCTD2 and KCTD10 in OC by overexpressing these genes in SKOV3 cells using expression vectors and assessing their impact on various tumorigenic behaviors. Upon successful transfection, RT‐qPCR analysis (Figures 9A and 10A) showed that KCTD2 and KCTD10 expression levels were significantly elevated in the overexpressed SKOV3 cells (OE‐KCTD2‐SKOV3 and OE‐KCTD10‐SKOV3) compared to control cells (Ctrl‐SKOV3). This increase in mRNA expression was further validated at the protein level using Western blot analysis (Figures 9B,C and 10B,C, and Figure S1), which confirmed higher KCTD2 and KCTD10 protein levels in the overexpressed cells, with GAPDH serving as a loading control. To assess the impact of KCTD2 and KCTD10 overexpression on cell proliferation, a CCK‐8 assay was performed (Figures 9D and 10D). The results demonstrated a significant reduction in cell proliferation in the OE‐KCTD2‐SKOV3 and OE‐KCTD10‐SKOV3 cells compared to controls, indicating that overexpression of these genes may inhibit ovarian cancer cell growth (Figures 9D and 10D). Further evaluation of the cells' ability to form colonies, which reflects their long‐term proliferative capacity, was conducted using a colony formation assay (Figures 9E,F and 10E–10F). The overexpression of KCTD2 and KCTD10 significantly decreased the number of colonies formed by SKOV3 cells (Figures 9E,F and 10D–F). To investigate the effect of KCTD2 and KCTD10 overexpression on cell migration, we performed a wound healing assay (Figures 9G–I and 10G–I). The results showed a marked reduction in wound healing percentage in OE‐KCTD2‐SKOV3 and OE‐KCTD10‐SKOV3 cells compared to control cells, indicating impaired migratory capabilities (Figures 9G,H and 10G,H).

**FIGURE 11:**
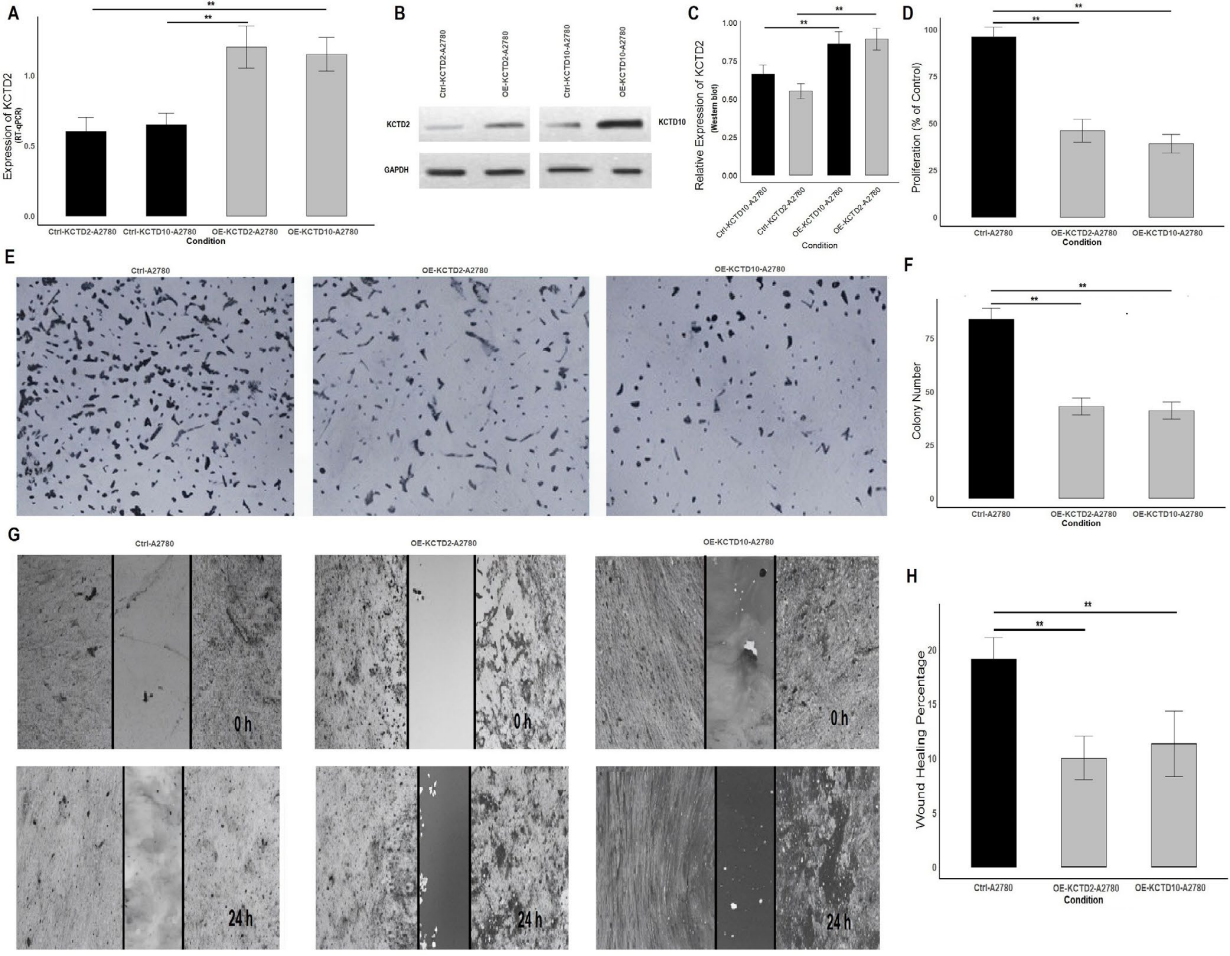
Validation of the tumor‐causing effects of KCTD2 and KCTD10 overexpression in the A2780 OC cell line. (A) RT‐qPCR analysis showing the expression levels of KCTD2 and KCTD10 in control (Ctrl‐A2780) and overexpression groups (OE‐KCTD2‐A2780 and OE‐KCTD10‐A2780). (B) Western blot analysis confirming increased protein expression of KCTD2 and KCTD10 in overexpressed A2780 cells, with GAPDH as the loading control. (C) Quantification of relative KCTD2 and KCTD10 expression from Western blot. (D) CCK‐8 assay results indicating reduced cell proliferation upon KCTD2 and KCTD10 overexpression. (E, F) Colony formation assay demonstrating a significant reduction in the number of colonies formed by OE‐KCTD2 and OE‐KCTD10 A2780 cells compared to control cells. (G, H) Wound healing assay showing impaired migration ability in A2780 cells overexpressing KCTD2 or KCTD10, as reflected by reduced wound closure percentage after 24 h. **p < 0.01.

The validation experiments conducted using the A2780 cell line further confirmed the tumor‐causing roles of KCTD2 and KCTD10 in OC. RT‐qPCR and Western blot analyses demonstrated that the overexpression of KCTD2 and KCTD10 significantly increased their mRNA and protein levels in overexpressed A2780 cells compared to controls (Figure 11A–C and Figure S1). Functional assays revealed that the overexpression of these genes markedly inhibited various oncogenic behaviors. The CCK‐8 assay showed a significant reduction in cell proliferation upon KCTD2 and KCTD10 overexpression in A2780 cells (Figure 11D), consistent with the findings observed in SKOV3 cells. Similarly, the colony formation assay demonstrated that OE‐KCTD2 and OE‐KCTD10 A2780 cells formed significantly fewer colonies than control cells (Figure 11E,F), indicating a reduction in their clonogenic potential. Furthermore, the wound healing assay revealed impaired migratory ability in A2780 cells overexpressing KCTD2 or KCTD10, as evidenced by a reduced wound closure percentage compared to control cells (Figure 11G,H).

## 4 Discussion

OC remains one of the deadliest gynecologic malignancies [8, 49, 50], characterized by late‐stage diagnosis, high recurrence rates, and poor survival outcomes [4, 51, 52]. Despite advancements in therapeutic strategies, the 5‐year survival rate for OC patients remains dismal due to a lack of reliable biomarkers for early diagnosis and prognosis [53, 54, 55]. Thus, identifying novel molecular targets is crucial for improving diagnosis, prognosis, and therapeutic interventions in OC [33, 56, 57]. The KCTD family proteins, known to be involved in diverse cellular processes such as ion channel regulation, protein degradation, and signal transduction, have garnered attention for their potential roles in cancer biology [58]. However, their implications in OC remain largely unexplored. In this study, we conducted a comprehensive analysis of the KCTD family genes in OC, evaluating their diagnostic, prognostic, and functional significance, along with their potential as therapeutic targets.

Our study revealed distinct expression patterns of KCTD genes across OC cell lines, indicating their potential as diagnostic biomarkers. Specifically, KCTD5, KCTD9, KCTD12, and KCTD16 were significantly upregulated, while KCTD2, KCTD9, KCTD10, KCTD15, and KCTD21 were downregulated in OC cell lines compared to normal controls. KCTD proteins are evolutionarily conserved and are generally categorized into clusters based on their sequence homology, which extends beyond the BTB (Broad‐Complex, Tramtrack, and Bric‐à‐brac) domain. This structural similarity suggests that members within the same cluster may exhibit overlapping biochemical functions and regulatory roles in cellular processes [59, 60]. Notably, KCTD2, KCTD5, and KCTD17 belong to the same evolutionary cluster, and their involvement in tumorigenesis has been increasingly recognized. KCTD5, for instance, has been implicated in protein degradation pathways by acting as a substrate adaptor for CUL3‐RING E3 ligases, facilitating the ubiquitination of key regulatory proteins involved in cell cycle control and apoptosis [61]. Conversely, KCTD2 has been linked to epigenetic regulation, including chromatin remodeling and transcriptional repression, which may contribute to tumor suppression in certain contexts [19, 62]. The dysregulated expression of these genes in OC suggests a complex interplay wherein certain members of the KCTD family may either functionally compensate for or antagonize each other's roles, thereby influencing cancer progression. The functional redundancy or divergence within this cluster highlights the need for further mechanistic studies to decipher their precise oncogenic or tumor‐suppressive roles in OC.

Importantly, while KCTD5 has been shown to interact with CUL3, literature reports suggest that KCTD12 and KCTD16 do not directly participate in ubiquitin‐mediated degradation pathways but instead interact with the GABAB2 receptor [19, 63]. These proteins function as auxiliary subunits of the GABAB receptor complex, modulating neuronal signaling rather than proteasomal degradation [64]. The upregulation of KCTD12 and KCTD16 in OC raises intriguing questions regarding their potential oncogenic functions beyond their known roles in neurotransmission. While evidence suggests that aberrant GABAergic signaling can influence cancer cell proliferation and migration, further studies are necessary to determine whether KCTD12 and KCTD16 contribute to OC pathogenesis via alternative mechanisms, such as modulating intracellular calcium homeostasis or interacting with non‐neuronal signaling pathways [65, 66]. The observed variability in KCTD gene expression highlights their functional complexity in cancer. Although structurally related proteins often exhibit similar biochemical activities, their expression patterns in OC suggest distinct or even opposing roles. For example, KCTD17 has been reported to modulate Notch and Wnt signaling, pathways that are crucial in OC proliferation and metastasis [23]. The potential redundancy or antagonism among KCTD members underscores the necessity for additional studies to elucidate their molecular interactions and contributions to tumorigenesis. Future research should investigate the crosstalk between KCTD proteins and major oncogenic pathways, the role of ubiquitination‐independent mechanisms in OC, and the therapeutic potential of targeting specific KCTD members to disrupt tumor‐promoting processes.

Our expression analysis findings are consistent with the existing literature, where KCTD12 has been previously reported to be upregulated in various cancers, including gastric and breast cancers, contributing to tumorigenesis by promoting cell survival and proliferation [67, 68, 69, 70]. Prior studies have indicated that KCTD5 is involved in ubiquitination processes that regulate cell cycle progression, which may explain its overexpression in OC and its role in promoting tumor growth [71, 72]. Moreover, our finding of decreased expression of KCTD2 and KCTD10 aligns with reports in glioma, where reduced levels of these genes were associated with tumor progression [23, 73]. Furthermore, the ROC analysis highlighted KCTD2, KCTD5, KCTD9, and KCTD12 as potential diagnostic markers with high AUC values (> 0.89), suggesting robust diagnostic accuracy for OC. Previous studies have primarily focused on the role of KCTD genes in neurodevelopmental disorders and a few cancer types, such as KCTD12 in gastrointestinal and breast cancers [74, 75], where it was reported to influence tumor progression and cell signaling pathways. In contrast, our findings highlight the potential of KCTD2, KCTD5, and KCTD9—genes with limited prior association with OC—as novel diagnostic indicators. Notably, while KCTD12 has been linked to poor prognosis in other malignancies [74, 76], its high diagnostic accuracy in OC revealed in our study represents a novel contribution.

Our study also analyzed the expression patterns of KCTD genes across different stages of OC. Notably, KCTD2, KCTD5, KCTD15, and KCTD21 showed significant stage‐dependent expression, suggesting a possible role in OC progression. Previous studies have linked KCTD5 to advanced cancer stages and poor prognosis in colorectal cancer and breast cancers [77, 78]. However, the associations of KCTD2, KCTD15, and KCTD21 with cancer progression remain underexplored, making our findings novel and significant. The mutational analysis revealed that while the overall mutation frequency of KCTD genes was low, specific missense mutations in KCTD12 and KCTD10 may have functional implications that warrant further investigation.

The promoter methylation analysis indicated a significant negative correlation between the methylation levels of KCTD genes and their mRNA expression in OC. Interestingly, hypomethylation of KCTD12 was associated with poor survival outcomes, including disease‐specific and overall survival, suggesting its potential as a prognostic biomarker. These findings are consistent with the notion that promoter hypomethylation may drive oncogene activation in cancers [79, 80]. To further explore the functional relevance of KCTD family genes in OC, we overexpressed KCTD2 and KCTD10 in SKOV3 and A2780 cells. Our results demonstrated that overexpression of these genes significantly reduced cell proliferation, colony formation, and migration, indicating tumor‐causing roles.

Previous studies have shown that miR‐183‐5p and miR‐9‐5p act as oncogenic miRNAs in OC, promoting proliferation, invasion, and poor prognosis, which aligns with their predicted regulation of KCTD genes [81, 82]. Similarly, miR‐218‐5p has been reported to have dual roles in cancer, supporting its potential context‐dependent function in OC [83]. In contrast, miR‐29c‐3p and miR‐199b‐3p are generally considered tumor suppressors [84, 85], and their upregulation in our context contrasts with prior findings, suggesting possible OC‐specific regulatory dynamics. Limited evidence on miR‐4500 and miR‐31‐5p in OC emphasizes the novelty of their proposed involvement and the need for further investigation.

Concerning merits and demerits, the strengths of this study lie in its comprehensive approach, combining RT‐qPCR validation, bioinformatics analysis from multiple databases, and functional assays to explore the diagnostic and prognostic potential of KCTD genes in OC. Our findings provide novel insights into the roles of these genes in OC, highlighting their impact on tumor behavior and patient outcomes. However, our study has certain limitations. Firstly, the reliance on OC cell lines may not fully replicate the tumor microenvironment and heterogeneity observed in clinical settings. Secondly, our use of publicly available databases, while valuable for large‐scale analyses, may not entirely capture the molecular complexities of OC in diverse patient populations. Additionally, we did not assess the potential correlation between KCTD2/KCTD10 and BRCA gene expression, which plays a crucial role in DNA repair and tumor progression in OC [86, 87]. Given BRCA's significance in OC proliferation and therapeutic response, future research should investigate whether BRCA expression modulates the tumor‐suppressive effects of KCTD2 and KCTD10. To address these limitations, future studies should incorporate primary OC tissue samples for validation and utilize in vivo models to explore mechanistic interactions. Moreover, transcriptomic and proteomic analyses should be conducted to examine potential regulatory links between BRCA and KCTD2/KCTD10, providing a more comprehensive understanding of their roles in OC progression.

## 5 Conclusion

In conclusion, this study offers a comprehensive analysis of the KCTD gene family in OC, uncovering their potential as novel diagnostic, prognostic, and therapeutic biomarkers. The differential expression of KCTD genes between OC and normal tissues, along with their association with patient survival outcomes, emphasizes their clinical relevance. Moreover, the observed links between specific KCTD members and key oncogenic processes—such as immune regulation, cell proliferation, and drug resistance—suggest that they may play critical roles in OC progression and treatment response. These findings not only enhance our understanding of the molecular landscape of OC but also pave the way for the development of targeted therapeutic strategies. Future investigations should focus on the functional characterization of individual KCTD genes, mechanistic studies to elucidate their roles in tumor biology, and validation of their biomarker potential in larger, independent patient cohorts and in vivo models.

## Author Contributions

**Ling Zhang:** investigation, writing – original draft, methodology, validation, visualization, software, formal analysis, data curation. **Chong Cheng:** investigation, writing – original draft, writing – review and editing, validation, visualization, methodology, data curation. **Bin Tang:** writing – original draft, writing – review and editing, conceptualization, supervision, data curation, formal analysis, project administration.

## Ethics Statement

The authors have nothing to report.

## Consent

The authors have nothing to report.

## Conflicts of Interest

The authors declare no conflicts of interest.

## Supporting information


**Data S1:** cam471147‐sup‐0001‐supinfo.pdf.

## Data Availability

Data would be provided by the corresponding author upon request.

## References

[1] Q. Lin , W. Ma , M. Xu , et al., “A Clinical Prognostic Model Related to T Cells Based on Machine Learning for Predicting the Prognosis and Immune Response of Ovarian Cancer,” Heliyon 10, no. 17 (2024): e36898. 39296051 10.1016/j.heliyon.2024.e36898 PMC11409031

[2] C. Bucur , S. Petrea , B. Gaspar , et al., “Ovarian Cancer Prevention and Screening–Where Do We Stand Today?,” Journal of Mind and Medical Sciences 11, no. 1 (2024): 99–105.

[3] Y. Meshkovska , A. Abramov , S. Mahira , and S. Thatikonda , “Understanding the Impact of Oxidative Stress on Ovarian Cancer: Advances in Diagnosis and Treatment,” (2024).

[4] A. Matsas , D. Stefanoudakis , T. Troupis , et al., “Tumor Markers and Their Diagnostic Significance in Ovarian Cancer,” Life 13, no. 8 (2023): 1689. 37629546 10.3390/life13081689 PMC10455076

[5] A. Koutras , P. Perros , I. Prokopakis , et al., “Advantages and Limitations of Ultrasound as a Screening Test for Ovarian Cancer,” Diagnostics 13, no. 12 (2023): 2078. 37370973 10.3390/diagnostics13122078 PMC10297553

[6] M. Khan and Y. Hameed , “Discovery of Novel Six Genes‐Based Cervical Cancer‐Associated Biomarkers That Are Capable to Break the Heterogeneity Barrier and Applicable at the Global Level,” Journal of Cancer Research and Therapeutics 9000 16 (2023): 738.

[7] Q. Zhu , J. Sun , C. An , et al., “Mechanism of LncRNA Gm2044 in Germ Cell Development,” Frontiers in Cell and Developmental Biology 12 (2024): 1410914. 39027044 10.3389/fcell.2024.1410914 PMC11255455

[8] N. Wu , X. Zhang , C. Fang , et al., “Progesterone Enhances Niraparib Efficacy in Ovarian Cancer by Promoting Palmitoleic‐Acid‐Mediated Ferroptosis,” Research 7 (2024): 371. 10.34133/research.0371 PMC11116976 38798714

[9] S. Zhan , F. Chen , L. Huang , et al., “The Clinical Pathological Characteristics and Prognostic Relevance of Homologous Recombination Repair Gene Mutations in Ovarian Cancer Patients: A Prospective Cohort Study,” Obstetrics and Gynecology International 2025, no. 1 (2025): 8247. 10.1155/ogi/5578247 PMC11957853 40166687

[10] V. Tavares , I. S. Marques , I. G. Melo , J. Assis , D. Pereira , and R. Medeiros , “Paradigm Shift: A Comprehensive Review of Ovarian Cancer Management in an era of Advancements,” International Journal of Molecular Sciences 25, no. 3 (2024): 1845. 38339123 10.3390/ijms25031845 PMC10856127

[11] A. Balaji , “Breaking Ground: Latest Innovations and Therapies in Ovarian Cancer Treatment,” Obstetrics and Gynaecology Forum 34, no. 2 (2024): 2024.

[12] M. A. Abdel‐Maksoud , S. Ullah , A. Nadeem , et al., “Unlocking the Diagnostic, Prognostic Roles, and Immune Implications of BAX Gene Expression in Pan‐Cancer Analysis,” American Journal of Translational Research 16, no. 1 (2024): 63–74. 38322551 10.62347/TWOY1681 PMC10839381

[13] Y. Hameed , “Decoding the Significant Diagnostic and Prognostic Importance of Maternal Embryonic Leucine Zipper Kinase in Human Cancers Through Deep Integrative Analyses,” Journal of Cancer Research and Therapeutics 19, no. 7 (2023): 1852–1864. 38376289 10.4103/jcrt.jcrt_1902_21

[14] V. M. Del Castillo Falconi , J. A. Godinez Rodriguez , V. Fragoso‐Ontiveros , et al., “Role of DNA Methylation and Non‐Coding RNAs Expression in Pathogenesis, Detection, Prognosis, and Therapy‐Resistant Ovarian Carcinoma,” Molecular Medicine Reports 31, no. 6 (2025): 144. 40183399 10.3892/mmr.2025.13509 PMC11979574

[15] S. Basu , R. Nadhan , and D. N. Dhanasekaran , “Long Non‐Coding RNAs in Ovarian Cancer: Mechanistic Insights and Clinical Applications,” Cancers 17, no. 3 (2025): 472. 39941838 10.3390/cancers17030472 PMC11815776

[16] J. Pang , N. Ding , X. Liu , et al., “Prognostic Value of the Baseline Systemic Immune‐Inflammation Index in HER2‐Positive Metastatic Breast Cancer: Exploratory Analysis of Two Prospective Trials,” Annals of Surgical Oncology 32, no. 2 (2025): 750–759. 39565489 10.1245/s10434-024-16454-8

[17] T. Bin , J. Tang , B. Lu , X.‐J. Xu , C. Lin , and Y. Wang , “Construction of AML Prognostic Model With CYP2E1 and GALNT12 Biomarkers Based on Golgi‐Associated Genes,” Annals of Hematology 103 (2024): 1–18. 39604595 10.1007/s00277-024-06119-7

[18] N. Balasco , L. Esposito , G. Smaldone , M. Salvatore , and L. Vitagliano , “A Comprehensive Analysis of the Structural Recognition Between KCTD Proteins and Cullin 3,” International Journal of Molecular Sciences 25, no. 3 (2024): 1881. 38339159 10.3390/ijms25031881 PMC10856315

[19] A. Angrisani , A. Di Fiore , E. De Smaele , and M. Moretti , “The Emerging Role of the KCTD Proteins in Cancer,” Cell Communication and Signaling 19, no. 1 (2021): 56. 34001146 10.1186/s12964-021-00737-8 PMC8127222

[20] Y.‐X. Shi , J.‐H. Yan , W. Liu , and J. Deng , “Identifies KCTD5 as a Novel Cancer Biomarker Associated With Programmed Cell Death and Chemotherapy Drug Sensitivity,” BMC Cancer 23, no. 1 (2023): 408. 37149576 10.1186/s12885-023-10895-2 PMC10163697

[21] Z. Liu , Y. Xiang , and G. Sun , “The KCTD Family of Proteins: Structure, Function, Disease Relevance,” Cell & Bioscience 3 (2013): 1–5. 24268103 10.1186/2045-3701-3-45 PMC3882106

[22] M. Skoblov , A. Marakhonov , E. Marakasova , et al., “Protein Partners of KCTD Proteins Provide Insights About Their Functional Roles in Cell Differentiation and Vertebrate Development,” BioEssays 35, no. 7 (2013): 586–596. 23592240 10.1002/bies.201300002

[23] X. Teng , A. Aouacheria , L. Lionnard , et al., “KCTD: A New Gene Family Involved in Neurodevelopmental and Neuropsychiatric Disorders,” CNS Neuroscience & Therapeutics 25, no. 7 (2019): 887–902. 31197948 10.1111/cns.13156 PMC6566181

[24] Y. Liao , D. C. Sloan , J. H. Widjaja , and B. S. Muntean , “KCTD5 Forms Hetero‐Oligomeric Complexes With Various Members of the KCTD Protein Family,” International Journal of Molecular Sciences 24, no. 18 (2023): 14317. 37762619 10.3390/ijms241814317 PMC10531988

[25] L. Pirone , G. Smaldone , R. Spinelli , et al., “KCTD1: A Novel Modulator of Adipogenesis Through the Interaction With the Transcription Factor AP2α,” Biochimica et Biophysica Acta 1864, no. 12 (2019): 158514. 31465887 10.1016/j.bbalip.2019.08.010

[26] R. Fjær , “Exome Sequencing in Monogenic Epilepsies. Genetic and Functional Studies of Monogenic Constitutional and Somatic Epilepsies,” (2024).

[27] J. Zhou , Z. Guo , X. Peng , et al., “Chrysotoxine Regulates Ferroptosis and the PI3K/AKT/mTOR Pathway to Prevent Cervical Cancer,” Journal of Ethnopharmacology 338 (2025): 119126. 39557107 10.1016/j.jep.2024.119126

[28] H. Zhang , C. Cao , and H. Xiong , “Construction and Validation of a Prognostic Model for Stemness‐Related Genes in Lung Adenocarcinoma,” Translational Cancer Research 13, no. 3 (2024): 1351–1366. 38617509 10.21037/tcr-23-1847 PMC11009808

[29] J. Suppiah , S. S. Md Sani , S. S. Hassan , et al., “Unraveling Potential Gene Biomarkers for Dengue Infection Through RNA Sequencing,” Virus Genes 1 (2024): 1–12. 10.1007/s11262-024-02114-2 PMC11787201 39397194

[30] H. Engqvist , T. Z. Parris , A. Kovács , et al., “Validation of Novel Prognostic Biomarkers for Early‐Stage Clear‐Cell, Endometrioid and Mucinous Ovarian Carcinomas Using Immunohistochemistry,” Frontiers in Oncology 10 (2020): 162. 32133296 10.3389/fonc.2020.00162 PMC7040170

[31] S. Wang , J. Fu , and X. Fang , “A Novel DNA Methylation‐Related Gene Signature for the Prediction of Overall Survival and Immune Characteristics of Ovarian Cancer Patients,” Journal of Ovarian Research 16, no. 1 (2023): 62. 36978087 10.1186/s13048-023-01142-0 PMC10053775

[32] F.‐C. Qian , L.‐W. Zhou , Y.‐Y. Li , et al., “SEanalysis 2.0: A Comprehensive Super‐Enhancer Regulatory Network Analysis Tool for Human and Mouse,” Nucleic Acids Research 51, no. W1 (2023): W520–W527. 37194711 10.1093/nar/gkad408 PMC10320134

[33] Q. Zeng , C. Chen , C. Chen , et al., “Serum Raman Spectroscopy Combined With Convolutional Neural Network for Rapid Diagnosis of HER2‐Positive and Triple‐Negative Breast Cancer,” Spectrochimica Acta Part A: Molecular and Biomolecular Spectroscopy 286 (2023): 122000. 36279798 10.1016/j.saa.2022.122000

[34] X. Ma , H. Cheng , J. Hou , et al., “Detection of Breast Cancer Based on Novel Porous Silicon Bragg Reflector Surface‐Enhanced Raman Spectroscopy‐Active Structure,” Chinese Optics Letters 18, no. 5 (2020): 51701.

[35] Z. Tang , B. Kang , C. Li , T. Chen , and Z. Zhang , “GEPIA2: An Enhanced Web Server for Large‐Scale Expression Profiling and Interactive Analysis,” Nucleic Acids Research 47, no. W1 (2019): W556–W560. 31114875 10.1093/nar/gkz430 PMC6602440

[36] G. Tang , M. Cho , and X. Wang , “OncoDB: An Interactive Online Database for Analysis of Gene Expression and Viral Infection in Cancer,” Nucleic Acids Research 50, no. D1 (2022): D1334–D1339. 34718715 10.1093/nar/gkab970 PMC8728272

[37] E. Cerami , J. Gao , U. Dogrusoz , et al., “The cBio Cancer Genomics Portal: An Open Platform for Exploring Multidimensional Cancer Genomics Data,” Cancer Discovery 2, no. 5 (2012): 401–404. 22588877 10.1158/2159-8290.CD-12-0095 PMC3956037

[38] C. J. Liu , F. F. Hu , G. Y. Xie , et al., “GSCA: An Integrated Platform for Gene Set Cancer Analysis at Genomic, Pharmacogenomic and Immunogenomic Levels,” Briefings in Bioinformatics 24, no. 1 (2023): bbac558. 36549921 10.1093/bib/bbac558

[39] A. Lánczky and B. Győrffy , “Web‐Based Survival Analysis Tool Tailored for Medical Research (KMplot): Development and Implementation,” Journal of Medical Internet Research 23, no. 7 (2021): 27633. 10.2196/27633 PMC8367126 34309564

[40] U. Karamat , S. Ejaz , and Y. Hameed , “In Silico‐Analysis of the Multi‐Omics Data Identified the Ataxia Telangiectasia Mutated Gene as a Potential Biomarker of Breast Invasive Carcinoma,” Genetic Testing and Molecular Biomarkers 25, no. 4 (2021): 263–275. 33877897 10.1089/gtmb.2020.0249

[41] S. J. Park , B. H. Yoon , S. K. Kim , and S. Y. Kim , “GENT2: An Updated Gene Expression Database for Normal and Tumor Tissues,” BMC Medical Genomics 12, no. Suppl 5 (2019): 19–514. 31296229 10.1186/s12920-019-0514-7 PMC6624177

[42] B. Ru , C. N. Wong , Y. Tong , et al., “TISIDB: An Integrated Repository Portal for Tumor‐Immune System Interactions,” Bioinformatics 35, no. 20 (2019): 4200–4202. 30903160 10.1093/bioinformatics/btz210

[43] H. Yuan , M. Yan , G. Zhang , et al., “CancerSEA: A Cancer Single‐Cell State Atlas,” Nucleic Acids Research 47, no. D1 (2019): D900–D908. 30329142 10.1093/nar/gky939 PMC6324047

[44] V. Agarwal , G. W. Bell , J. W. Nam , and D. P. Bartel , “Predicting Effective microRNA Target Sites in Mammalian mRNAs,” eLife 12, no. 4 (2015): 05005. 10.7554/eLife.05005 PMC4532895 26267216

[45] D. Szklarczyk , R. Kirsch , M. Koutrouli , et al., “The STRING Database in 2023: Protein‐Protein Association Networks and Functional Enrichment Analyses for Any Sequenced Genome of Interest,” Nucleic Acids Research 51, no. D1 (2023): D638–D646. 36370105 10.1093/nar/gkac1000 PMC9825434

[46] D. Warde‐Farley , S. L. Donaldson , O. Comes , et al., “The GeneMANIA Prediction Server: Biological Network Integration for Gene Prioritization and Predicting Gene Function,” Nucleic Acids Research 38 (2010): W214–W220. 20576703 10.1093/nar/gkq537 PMC2896186

[47] B. T. Sherman , M. Hao , J. Qiu , et al., “DAVID: A Web Server for Functional Enrichment Analysis and Functional Annotation of Gene Lists (2021 Update),” Nucleic Acids Research 50, no. W1 (2022): W216–W221. 35325185 10.1093/nar/gkac194 PMC9252805

[48] A. Jia , L. Xu , and Y. Wang , “Venn Diagrams in Bioinformatics,” Briefings in Bioinformatics 22, no. 5 (2021): bbab108. 33839742 10.1093/bib/bbab108

[49] L. Han , S. Xu , D. Zhou , et al., “Unveiling the Causal Link Between Metabolic Factors and Ovarian Cancer Risk Using Mendelian Randomization Analysis,” Frontiers in Endocrinology 15 (2024): 1401648. 38899007 10.3389/fendo.2024.1401648 PMC11185996

[50] X. Wang , X. Qian , D. Zhao , R. Xu , and Z. Liu , “Role of KIF20A Depletion in Inhibiting Ovarian Cancer Progression: Insights From PTEN and M2 Macrophage Polarization,” Discovery Medicine 36, no. 191 (2024): 2433–2444. 39726317 10.24976/Discov.Med.202436191.224

[51] M. Mehrotra , P. Phadte , P. Shenoy , S. Chakraborty , S. Gupta , and P. Ray , “Drug‐Resistant Epithelial Ovarian Cancer: Current and Future Perspectives,” Cell and Molecular Biology of Ovarian Cancer: Updates, Insights and New Frontiers 1 (2024): 65–96. 10.1007/978-3-031-58311-7_4 38805125

[52] F. Jiang , S. Ahmad , S. Kanwal , Y. Hameed , and Q. Tang , “Key Wound Healing Genes as Diagnostic Biomarkers and Therapeutic Targets in Uterine Corpus Endometrial Carcinoma: An Integrated In Silico and In Vitro Study,” Hereditas 162, no. 1 (2025): 5. 39833941 10.1186/s41065-025-00369-9 PMC11748876

[53] Y. Zhang and L. Tian , “Advances and Challenges in the Use of Liquid Biopsy in Gynaecological Oncology,” Heliyon 10 (2024): e39148. 39492906 10.1016/j.heliyon.2024.e39148 PMC11530831

[54] M. Zhang , R. Yin , and K. Li , “Advances and Challenges in the Origin and Evolution of Ovarian Cancer Organoids,” Frontiers in Oncology 14 (2024): 1429141. 39220646 10.3389/fonc.2024.1429141 PMC11362079

[55] M. A. Hegazi , F. Pasqualini , G. Taverna , R. S. Bresalier , M. Chiriva‐Internati , and F. Grizzi , “Investigating the Spatial Distribution of Proliferating Cells in Primary Ovarian Cancers,” Discovery Medicine 36, no. 182 (2024): 632–645. 38531804 10.24976/Discov.Med.202436182.60

[56] N. Sial , M. Ahmad , M. S. Hussain , et al., “CTHRC1 Expression Is a Novel Shared Diagnostic and Prognostic Biomarker of Survival in Six Different Human Cancer Subtypes,” Scientific Reports 11, no. 1 (2021): 19873. 34615943 10.1038/s41598-021-99321-w PMC8494806

[57] M. Usman , M. K. Okla , H. M. Asif , et al., “A Pan‐Cancer Analysis of GINS Complex Subunit 4 to Identify Its Potential Role as a Biomarker in Multiple Human Cancers,” American Journal of Cancer Research 12, no. 3 (2022): 986–1008. 35411239 PMC8984884

[58] L. Esposito , N. Balasco , G. Smaldone , R. Berisio , A. Ruggiero , and L. Vitagliano , “AlphaFold‐Predicted Structures of KCTD Proteins Unravel Previously Undetected Relationships Among the Members of the Family,” Biomolecules 11, no. 12 (2021): 1862. 34944504 10.3390/biom11121862 PMC8699099

[59] A. Bonchuk , K. Balagurov , and P. Georgiev , “BTB Domains: A Structural View of Evolution, Multimerization, and Protein–Protein Interactions,” BioEssays 45, no. 2 (2023): 2200179. 10.1002/bies.202200179 36449605

[60] T. Xing , J. Chen , J. Ding , J. Liu , S. Ling , and Y. Luo , “CircRNA Hsa_circ_0120175 Promotes Ovarian Cancer Tumorigenesis and Predicts a Poor Prognosis,” Discovery Medicine 36, no. 180 (2024): 113–120. 38273751 10.24976/Discov.Med.202436180.10

[61] M. Maekawa and S. Higashiyama , “KCTD10 Biology: An Adaptor for the Ubiquitin E3 Complex Meets Multiple Substrates: Emerging Divergent Roles of the Cullin‐3/KCTD10 E3 Ubiquitin Ligase Complex in Various Cell Lines,” BioEssays 42, no. 8 (2020): 1900256. 10.1002/bies.201900256 32484264

[62] G. Zhang , C. Song , M. Yin , et al., “TRAPT: A Multi‐Stage Fused Deep Learning Framework for Predicting Transcriptional Regulators Based on Large‐Scale Epigenomic Data,” Nature Communications 16, no. 1 (2025): 3611. 10.1038/s41467-025-58921-0 PMC12003887 40240358

[63] N. Balasco , L. Pirone , G. Smaldone , et al., “Molecular Recognition of Cullin3 by KCTDs: Insights From Experimental and Computational Investigations,” Biochimica et Biophysica Acta (BBA)‐Proteins and Proteomics 1844, no. 7 (2014): 1289–1298. 24747150 10.1016/j.bbapap.2014.04.006

[64] M. Terunuma , “Diversity of Structure and Function of GABAB Receptors: A Complexity of GABAB‐Mediated Signaling,” Proceedings of the Japan Academy. Series B, Physical and Biological Sciences 94, no. 10 (2018): 390–411. 30541966 10.2183/pjab.94.026 PMC6374141

[65] A. Nieto , T. Bailey , K. Kaczanowska , and P. McDonald , “GABAB Receptor Chemistry and Pharmacology: Agonists, Antagonists, and Allosteric Modulators,” Behavioral Neurobiology of GABAB Receptor Function 4 (2021): 81–118. 10.1007/7854_2021_232 34036555

[66] M. Ernst , “Effects of the Atypical Antipsychotic Clozapine on Recombinantly Expressed GABAA Receptor Subtypes zur Erlangung des Akademischen Grades,” Dr. Med. Univ.

[67] Z. Wang , D. Wu , M. Dong , Y. Xia , and T. Xu , “KCTD12 Is a Prognostic Marker of Breast Cancer and Correlates With Tumor Immune Cell Infiltration,” Translational Cancer Research 10, no. 1 (2021): 261–272. 35116258 10.21037/tcr-20-2099 PMC8798602

[68] Y. Ry , K. Xy , Z. Hj , N. Shao , Y. Lin , and W. Sm , “KCTD12 Promotes G1/S Transition of Breast Cancer Cell Through Activating the AKT/FOXO1 Signaling,” Journal of Clinical Laboratory Analysis 34, no. 8 (2020): e23315. 32207860 10.1002/jcla.23315 PMC7439418

[69] J.‐F. Wang and J.‐P. Niu , “KCTD‐12 Inhibits the in Vitro Proliferation and Invasion of Gastrointestinal Stromal Tumor Cells: The Experimental Study,” Journal of Hainan Medical University 23, no. 12 (2017): 8–11.

[70] D. Kubota , A. Yoshida , H. Tsuda , et al., “Gene Expression Network Analysis of ETV1 Reveals KCTD10 as a Novel Prognostic Biomarker in Gastrointestinal Stromal Tumor,” PLoS One 8, no. 8 (2013): e73896. 23977394 10.1371/journal.pone.0073896 PMC3747077

[71] J. Canales , P. Cruz , N. Díaz , D. Riquelme , E. Leiva‐Salcedo , and O. Cerda , “K+ Channel Tetramerization Domain 5 (KCTD5) Protein Regulates Cell Migration, Focal Adhesion Dynamics and Spreading Through Modulation of Ca2+ Signaling and Rac1 Activity,” Cells 9, no. 10 (2020): 2273. 33053687 10.3390/cells9102273 PMC7600296

[72] M. P. Saldías Maulén , “Effect of SARAF‐Based Peptides on the Store‐Operated Calcium Entry and Cell Invasion in Triple‐Negative Breast Cancer Model,” (2023).

[73] A. Birerdinc , Role of KCNRG in B‐Cell Chronic Lymphocytic Leukemia (George Mason University, 2008).

[74] P. Liu , Z. Liu , Q. Luo , et al., “A Pan‐Cancer Analysis of Potassium Channel Tetramerization Domain Containing 12 in Human Cancer,” Scientific Reports 13, no. 1 (2023): 13898. 37626178 10.1038/s41598-023-41091-8 PMC10457314

[75] W. Shen , Y. Li , B. Li , et al., “Downregulation of KCTD12 Contributes to Melanoma Stemness by Modulating CD271,” Cancer Biology & Medicine 16, no. 3 (2019): 498–513. 31565480 10.20892/j.issn.2095-3941.2019.0073 PMC6743620

[76] L. Luo , Q. Zhao , X. Cui , et al., “Male‐Specific Lethal 1 (MSL1) Promotes Erastin‐Induced Ferroptosis in Colon Cancer Cells by Regulating the KCTD12‐SLC7A11 Axis,” Cell Death & Disease 16, no. 1 (2025): 281. 40221412 10.1038/s41419-025-07555-7 PMC11993775

[77] J. Rivas , N. Díaz , I. Silva , et al., “KCTD5, a Novel TRPM4‐Regulatory Protein Required for Cell Migration as a New Predictor for Breast Cancer Prognosis,” FASEB Journal 34 (2020): 7847–7865. 32301552 10.1096/fj.201901195RRR

[78] H. Yao , D. Ren , Y. Wang , et al., “KCTD9 Inhibits the Wnt/β‐Catenin Pathway by Decreasing the Level of β‐Catenin in Colorectal Cancer,” Cell Death & Disease 13, no. 9 (2022): 761. 36055981 10.1038/s41419-022-05200-1 PMC9440223

[79] A. Van Tongelen , A. Loriot , and C. De Smet , “Oncogenic Roles of DNA Hypomethylation Through the Activation of Cancer‐Germline Genes,” Cancer Letters 396 (2017): 130–137. 28342986 10.1016/j.canlet.2017.03.029

[80] Y.‐C. Liu , J. Kwon , E. Fabiani , et al., “Demethylation and Up‐Regulation of an Oncogene After Hypomethylating Therapy,” New England Journal of Medicine 386, no. 21 (2022): 1998–2010. 35613022 10.1056/NEJMoa2119771 PMC9514878

[81] M. Flores‐Colon , M. Rivera‐Serrano , V. G. Reyes‐Burgos , et al., “MicroRNA Expression Profiles in Human Samples and Cell Lines Revealed Nine miRNAs Associated With Cisplatin Resistance in High‐Grade Serous Ovarian Cancer,” International Journal of Molecular Sciences 25, no. 7 (2024): 3793. 38612604 10.3390/ijms25073793 PMC11011404

[82] F. Davodabadi , S. Mirinejad , S. Malik , et al., “Nanotherapeutic Approaches for Delivery of Long Non‐Coding RNAs: An Updated Review With Emphasis on Cancer,” Nanoscale 16 (2024): 3881–3914. 38353296 10.1039/d3nr05656b

[83] J. C. Kovacic , S. Dimmeler , R. P. Harvey , et al.,“Endothelial to Mesenchymal Transition in Cardiovascular Disease: JACC State‐of‐the‐Art Review,” J Am Coll Cardiol 73, no. 2 (2019): 190–209. 30654892 10.1016/j.jacc.2018.09.089 PMC6865825

[84] Q. Wang , B. Ye , P. Wang , F. Yao , C. Zhang , and G. Yu , “Overview of microRNA‐199a Regulation in Cancer,” Cancer Management and Research 11 (2019): 10327–10335. 31849522 10.2147/CMAR.S231971 PMC6911337

[85] J. Liu , Z. Quan , Y. Gao , X. Wu , and Y. Zheng , “MicroRNA‐199b‐3p Suppresses Malignant Proliferation by Targeting Phospholipase Cε and Correlated With Poor Prognosis in Prostate Cancer,” Biochemical and Biophysical Research Communications 576 (2021): 73–79. 34482026 10.1016/j.bbrc.2021.08.078

[86] Y.‐X. Shi , W.‐D. Zhang , P.‐H. Dai , J. Deng , and L.‐H. Tan , “Comprehensive Analysis of KCTD Family Genes Associated With Hypoxic Microenvironment and Immune Infiltration in Lung Adenocarcinoma,” Scientific Reports 12, no. 1 (2022): 9938. 35705627 10.1038/s41598-022-14250-6 PMC9200823

[87] D. Senft , J. Qi , and Z. A. Ronai , “Ubiquitin Ligases in Oncogenic Transformation and Cancer Therapy,” Nature Reviews Cancer 18, no. 2 (2018): 69–88. 29242641 10.1038/nrc.2017.105 PMC6054770

