# Supplementary figures and images for: Mechanistic Insights Into the Tumor‐Driving and Diagnostic Roles of KCTD Family Genes in Ovarian Cancer: An Integrated In Silico and In Vitro Analysis
Source: Cancer Med. 2025 Aug 20;14(16):e71147. doi: 10.1002/cam4.71147 (PMC12365668; doi:10.1002/cam4.71147)

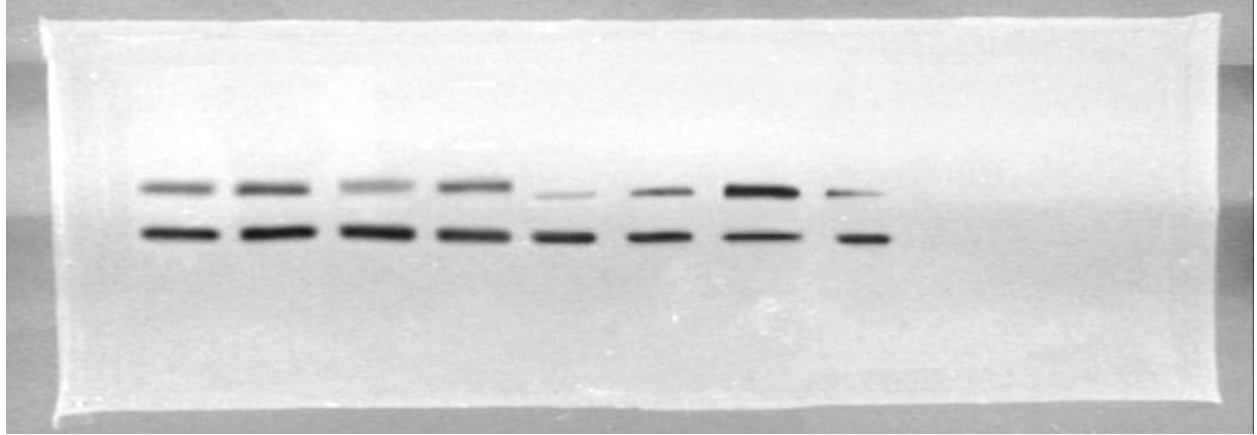

**Supplementary data Figure 1: Uncut Western blot bands of KCTD2, KCTD10 and GAPDH in SKOV-3 and A2780 cells.**

Supplement: Supplementary file 1 — Data S1: cam471147‐sup‐0001‐supinfo.pdf. [file CAM4-14-e71147-s001.pdf]
